# Examining the relationships between walkability and physical activity among older persons: what about stairs?

**DOI:** 10.1186/s12889-018-5945-0

**Published:** 2018-08-17

**Authors:** Nancy Edwards, Joshun Dulai

**Affiliations:** 1 School of Nursing, University of Ottawa, 1 Stewart Street Room 205, Ottawa, ON K1H8M5 Canada; 2 School of Nursing, University of Ottawa, 1 Stewart Street, Room 127, Ottawa, ON K1N 7M9 Canada

**Keywords:** Walkability, Physical activity, Older persons, Built environment, Stairs

## Abstract

**Background:**

Walkability is considered an important dimension of healthy communities. However, variable associations between measures of walkability and physical activity have been observed, particularly among older persons. Given the challenges older persons may have navigating stairs on walking routes, the presence of stairs may be an explanatory factor for these mixed associations. The purposes of this scoping review were to determine whether studies examining the relationship between walkability and physical activity included items that assessed stairs and what relationships were found.

**Methods:**

Systematic reviews were identified by entering search terms into five database search engines. Eligibility criteria were: a) published between 2008 and 2017, b) examined the relationship between walkability and physical activity, c) included a focus on persons aged 65 years and older, and d) written in English. The full articles for all primary studies included in eligible systematic reviews were then retrieved. Duplicates were removed. Information about where the study took place, walkability measures used, types of walkability data obtained (objective and/or subjective) and questions asked about stairs were extracted from the full text articles.

**Results:**

Eleven systematic reviews were identified; seven were eligible. After removing duplicates, 289 primary studies remained for review. Measures of neighborhood walkability were present in 205 studies; a minority (*n* = 5, 2.4%) included items about stairs. No information was obtained on the structural features of the stairs.

**Conclusions:**

The presence of stairs may deter older persons (and others) from walking outdoors. Standard measures to document the presence and characteristics of stairs, and sampling approaches to select stairs for assessment are needed. The inclusion of these measures would augment the utility and comparability of studies examining relationships between walkability and physical activity and better inform planning and policy decisions.

**Electronic supplementary material:**

The online version of this article (10.1186/s12889-018-5945-0) contains supplementary material, which is available to authorized users.

## Background

Walkability is considered an important dimension of healthy communities. Typically, walkability indices examine land use mix, street connectivity, and population density [1]. These indices are useful for intersectoral planning by public health departments, as the indices have their origins in, and thus resonate with actors in other sectors – notably urban planning and transportation [2].

Walkability indices have been used in community assessment and urban planning processes [3–5]. They are referred to in some guidelines for public health [6, 7] and used to rank cities [8, 9]. These indices started to gain prominence in public health over a decade ago, as issues around physical inactivity and obesity came to the fore. Walkable environments were thought to yield potential health benefits, both directly, by fostering more physical activity; and indirectly, by increasing active transportation and reducing urban air pollution. More walkable neighbourhoods have also been associated with stronger neighbourhood cohesion and connectedness [10], less social isolation [11], and less crime [12]. Yet, there are some concerns about walkability as a construct, particularly as it applies to older persons (defined as adults aged 65 years and older for the remainder of this article). Van Cauwenberg et al. [13] contrasted the relationships found between the built environment and physical activity among children and younger adults versus older persons. They described the latter association as weaker and more variable and suggested that more detailed assessments of the physical environment are required to better understand its relationship with physical activity among older persons. While environmental features such as walkability, street connectivity, sidewalk coverage, and esthetics were assessed in many of their 31 included studies, there was only a single mention of stairs in their review. Stairs may be a pertinent environmental feature, and especially for older persons, because difficulty navigating stairs is often described as an impediment by those experiencing mobility limitations [14–16]. Walking for leisure is a common form of physical activity among older adults [17] and thus encountering stairs along a walking route may be particularly troublesome for this population. In addition, reports of and checklists to guide the development of age-friendly neighbourhoods and cities stress the importance of designing safe stairs along with other features of the built environment such as curbs, benches, and lighting [18, 19]. However, it is indoor [20, 21] and outdoor stairs [22, 23], rather than the latter features that are associated with a high incidence of injurious falls among older persons. Furthermore, a fear of falling may limit physical activity in this age group [24–26].

There are a number of plausible explanations for the differences van Cauwenberg et al. [13] reported regarding relationships between the built environment and walkability in younger versus older populations. First, geographic measures may fail to capture local variations in environmental characteristics, which influence how and whether older persons are able to or choose to navigate their outdoor environment. Feuillet et al. [27] explored this in their study of spatial heterogeneity and active commuting in Paris. They concluded that socio-ecological measures need to be area specific. Pliakas et al. [28] compared foot-based street audits, virtual street audits and routinely collected data, noting the varying spatial scales depicted by the three methods. Similarly, in a review article, Rollings et al. [29] identified the importance of using the appropriate spatial scale in combination with physical measures of neighbourhoods that might moderate or mediate relationships between the neighbourhood environment and health. Second, subjective perceptions and objective measures of environmental characteristics yield different insights into what influences behaviours [29–32]. Subjective perceptions take into account one’s ease or difficulty in navigating the environment based on various personal attributes: physical, social or mental health; fear of falling; one’s confidence in being able to navigate the environment and one’s perspective of potential benefits. In Orstad’s review [32], comparing objective versus subjective measures, authors concluded that “the perceived neighborhood environment and objectively measured neighborhood environment are related but distinct constructs” and this distinction “accounts for unique variance in physical activity”. Third, the person-environment fit hypothesis [1, 33, 34] suggests that built environmental characteristics such as physical obstacles interact with physical limitations such as chronic disease or mobility restrictions [13]; to affect the relationships between walkability and health status.

Authors looking at relationships between walkability or the built environment and physical activity have taken some of these factors into account. For example, some have adjusted for confounders such as physical ability, socio-economic status (SES) [29, 35], age and gender [13] while others have added measures of environmental enablers or barriers for more robust covariate analyses [1]. Other authors [30] have provided solid methodological critiques of literature examining relationships between the built environment and physical activity. However, our preliminary examination of the afore-mentioned studies indicated that stairs on walking paths were not described as environmental factors, despite the fact that stairs are often described as a navigational challenge for older persons [36].

Therefore, the purposes of this scoping review were to determine whether studies examining the relationship between walkability and physical activity included items that assessed stairs and what relationships were found.

## Methods

### Literature search

We followed those guidelines in the PRISMA (Preferred Reporting Items for Systematic Review and Meta-Analysis) statement [37] that were pertinent to a scoping review. We used a two-step process to identify articles, starting with systematic reviews and then primary studies included in those reviews. Our focus was on systematic reviews conducted in the last decade. First, we identified all systematic reviews by entering the search terms “walkability”, “physical activity”, and “systematic review” or “review” into the following database search engines: PubMed, CINAHL, and OVID (Medline, EMbase, PsycINFO) databases. Reviews were included if they were published within the last 10 years (2008–2017), examined the relationship between walkability and physical activity, included a focus on older persons, and were written in English. Eligibility was assessed by reviewing titles and abstracts. Eleven reviews were retrieved; seven were deemed eligible after review by both authors. Primary eligible studies included in each review (regardless of their publication date) were then identified by reviewing tables and supplementary materials of said reviews. Duplicates were removed by examining titles and abstracts.

The full text of all primary studies were retrieved. When these published studies reported that data about walkability was obtained but information about the actual questionnaire, index or assessment tools used was missing, we attempted to retrieve this missing information from other supplementary sources using the search engines Google and Google Scholar.

Data extracted from systematic reviews were: author and year of publication; number of primary studies included; definition of walkability used; age criteria applied for inclusion of primary studies; whether or not a methodological quality assessment of primary studies was done; outcome variables assessed; main findings describing the relationships between walkability and physical activity; any mention of geographic location (i.e., urban, rural, etc.) or terrain (i.e. slopes, hills, etc.); and any mention of stairs in methods, results or discussion sections. Data extracted from primary studies included: author and year of publication, country (ies) where study undertaken, age of participants, walkability measure included (yes or no), walkability measure used, type of walkability data obtained (objective and/or subjective), and stairs (defined as 2 or more steps) assessed in the study (yes or no and description of measure). Subjective measures of walkability were defined as those for which participants were asked to provide their own perceptions of neighbourhood walkability. Objective measures were defined as those for which the researchers determined the walkability of neighbourhoods using information from external sources (for example, Geographic Information Systems). For some primary studies, supplementary documents were reviewed to determine the type of walkability measure (objective or subjective) and whether or not stairs were assessed. When walkability measures could not be retrieved or were not available in English, responses to the foregoing were categorized as not available.

## Results

**Fig. 1:**
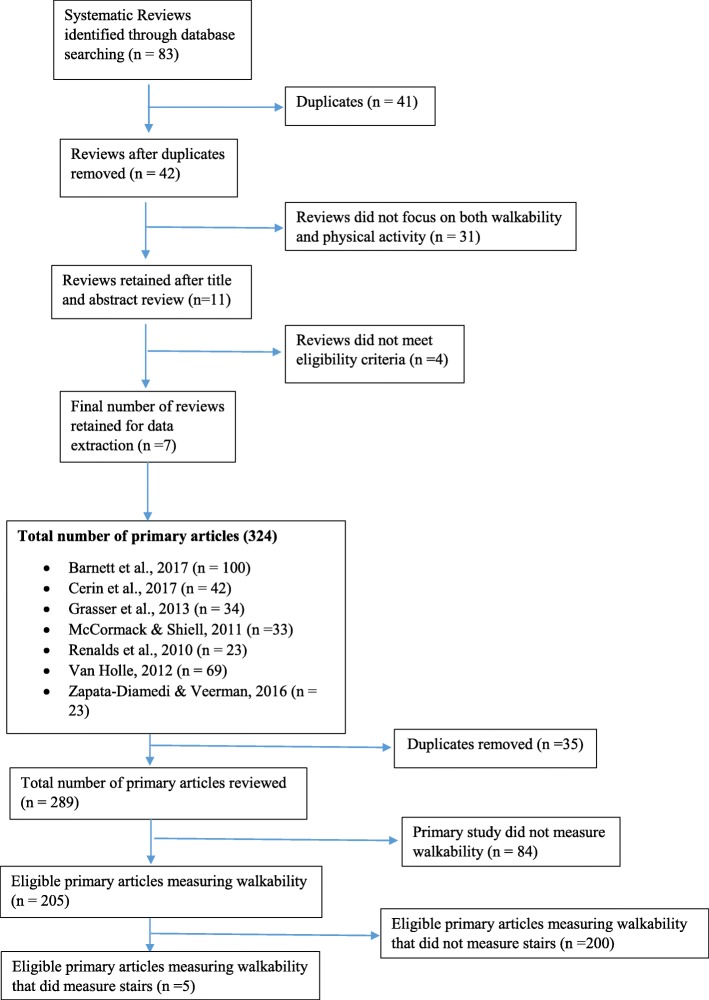
Study Retrieval Algorithm

The number of articles identified, retrieved and deemed eligible through our search strategy is shown in Fig. 1. The seven eligible systematic reviews yielded 289 primary studies (after duplicates were removed), which had been undertaken in over 33 countries (See Table 1 and, Additional file 1: Table S1). Just over two thirds (*n* = 205, 70.9%) of these primary studies included a measure of walkability.

**Table 1:** Summary of systematic reviews on walkability and physical activity included

Author (s) (Year)	Defined Walkability		Age Was a Criterion for Eligibility		Methodological Quality of Primary Studies Assessed		Outcome Variables	Findings of Interest
	No	Yes	No	Yes	No	Yes		
Barnett et al. (2017) [38]	✓			✓(65+)		✓	Total physical activity and total walking	Neighbourhood walkability was positively associated with older persons’ total physical activity and total walking.
Cerin et al. (2017) [39]		✓		✓(65+)		✓	Active travel (total walking for transport, within-neighbourhood walking for transport, cycling for transport, combined walking and cycling for transport, & all active travel outcomes combined)	Walkability was significantly positively associated with total walking and all active travel, positively associated with walking and cycling combined, but not associated with cycling for transport.
Grasser et al. (2013) [30]		✓		✓		✓	Walking and cycling for transport, overall active transportation, and weight-related measures	Walkability measures were consistently positively associated with walking in studies examined. Correlations between overall active transportation and weight-related measures were weak.
McCormack & Shiell (2011) [42]	✓		✓		✓		Physical activity	Walkability indices and land-use mix (a component of walkability) were consistently positively associated with physical activity, after controlling for neighbourhood self-selection.
Renalds et al. (2010) [43]	✓		✓		✓		Health (physical activity, obesity and overweight, social capital, and mental health)	More walkable neighbourhoods were associated with higher levels of physical activity among residents.
Van Holle (2012) [40]		✓		✓(18–65)	✓		Physical activity domains: total physical activity, leisure-time physical activity, total walking and/or cycling, recreational walking and/or cycling, active transportation in general, transportation walking, and transportation cycling	Overall walkability was positively associated with total physical activity, transportation walking, and transportation cycling across studies. It was not associated with recreational or leisure time physical activity. There weren’t enough studies to make any conclusions about the relationship between walkability with total walking/cycling or general active transportation
Zapata-Diomedi & Veerman (2016) [41]	✓			✓(18+)		✓	Physical activity	Strong positive associations between physical activity and walkability overall. Stronger associations between walkability and transport-related and total physical activity than between walkability and recreational physical activity.

Two reviews focused solely on older persons [38, 39], three reviews included younger adults [30, 40, 41], while the remaining two reviews did not use age as an eligibility criterion [42, 43]. Four reviews assessed the quality of primary studies [30, 38, 39, 41]. Three of the seven reviews provided an explicit definition for walkability [38, 42, 43]. All of these definitions incorporated land use mix diversity, street connectivity, and residential density as definitional components. All reviews reported on the types of walkability measures used as well as other environmental features recorded in primary studies. All described associations found between walkability and physical activity, although there was considerable variation in how authors examined similarities and/or inconsistencies in findings among primary studies. None of the reviews provided any mention of stairs in their methods, results or discussion sections.

Five of the seven reviews also referred to the geographic location and terrain in some of the primary studies they examined [38–40, 42]. With regards to geographic location, urbanization was a variable for Van Holle et al. [40] and comparisons of urban settings versus rural settings were examined by Barnett and Cerin [38, 39]. In addition, two primary studies examined by McCormack [42] focused on urban vs suburban comparisons [44, 45] whereas participants in all the studies examined by Grasser et al. lived in urban and/or suburban environments [30]. With regards to geographic terrain, “hilliness” was a variable of interest in Van Holle et al. [40] while Cerin [39] looked at sloping streets as a moderator between physical environmental correlates of active travel in older adults. Topography was included as part of a neighbourhood pedestrian environment score in a study examined by McCormack et al. [46] and the absence of physical environmental barriers, such as hills, was examined by Barnett et al. in their review [38]. In the background for their paper, Barnett et al. also described physically-challenging environments as those that included inclines and uneven surfaces. In addition, Tanaka et al. [47] compared the physical activity of community-dwelling older women living in sloped versus non-sloped environments.

A couple of findings stand out from these reviews. First, there were more consistent and positive relationships reported between walkability and physical activity than between walkability and either active transportation or health status. For example, Grasser et al. [30] reported that the walkability measures of gross population density, housing unit density, and intersection density were consistently associated with more walking among participants. However, associations between connectivity measures and walking for transport were inconsistent. They concluded that the weak and inconsistent correlations between walkability and physical activity for active transport were due to study design (most studies were cross-sectional) and the quality of publications (most were rated as low or fair quality). Second, there were no obvious patterns across reviews in either the moderating variables described and/or in their moderating effects. For example, in the systematic review by Cerin et al. [39], there were no significant moderating effects for age, driving status, area-level household income, area-level SES, traffic safety, pedestrian safety, and crime safety between walkability and (total) walking. Barnett et al. [38] identified 16 moderators in their review of 39 articles. They found inconsistencies in the direction of effects in significant interaction terms.

The majority of the primary studies examined by these reviews included older persons as participants (247/289, 85.4%) with over a quarter of these studies focusing exclusively on this population (71/247, 28.7%). Only 4.5% (13/289) of the primary studies did not include older adults as participants, with the inclusion of older adults in the remaining studies (30/289, 10.4%) being difficult to confirm.

Among the 205 primary studies that included a measure of walkability, 14 different standard measures that directly or indirectly assess walkability were described (See Table 2). Authors of 45 studies (22.0%) reported developing their own questionnaire items or indices of walkability; 32 of these authors exclusively used these non-standard measures to assess walkability. The most commonly used source of walkability data was a geographic information system (GIS); 110 studies (53.7%) reported this type of objective data source. The second most commonly used measure was the Neighborhood Environment Walkability Scale (NEWS) with 51 (24.9%) articles citing this subjective measure.

**Table 2:** Measures of walkability: tools, indices and questionnaires and stair assessment

Name/Type of walkability tool, index, or questionnaire (*N* = 15)	Assesses Geographic Terrain	Assesses Stairs	Number of Articles Using This Measure (%)
Audit of Physical Activity Resources for Seniors (APARS)	Yes	Yes	1 (0.5%)
Geographic Information Systems (GIS)	Yes^1^	No	110 (53.7%)
International Physical Activity Questionnaire Environmental Module (IPAQ-E)	No	No	5 (2.4%)
Neighborhood Brief Observation Tool	No	No	1 (0.5%)
Neighborhood Environment Walkability Scale (NEWS; all versions)	Yes	No	51 (24.9%)
Neighborhood Open Space (NOS)	No	No	3 (1.5%)
Neighborhood Resident Survey	No	No	1 (0.5%)
Neighborhood Walking Questionnaire for Chinese Seniors (NWQ-CS)	Yes	No	4 (2.0%)
New Urbanism Index	No	No	1 (0.5%)
Older Peoples Active Living (OPAL) questionnaire	Unknown	Unknown	3 (1.5%)
Self-created items or indices on walkability	Yes^a^	Yes^a^	45 (22.0%)
(Street Smart) Walk Score	No	No	5 (2.4%)
Systematic Pedestrian and Cycling Environment Scan (SPACES)	Yes	No	1 (0.5%)
University of Miami Built Environment Coding System (UMBECS)	No	No	1 (0.5%)
Zhongshan Household Travel Survey (ZHTS)	Unknown	Unknown	1 (0.5%)
Total	6	1^b^	205 (100%)^c^

^a^Refers to a broader set of walkability measures and thus some collect information on geographic terrain while others do not

^b^APARS is the only walkability tool that that includes questions on stairs. However, in four other articles there were questions about stairs as survey items in their studies about walkability

^c^Since some studies used more than one type of walkability measure, the numbers and percentages add up to more than 205 and 100% respectively

Only four authors reporting on five studies (2.4% of the 205 primary studies with walkability indices) included any questions about stairs [48–52]. As shown in Table 3, these questions were very limited in scope and variable in content. One author asked about stairs at work [48], another about stairs in the neighbourhood [51], and the others about stairs outside their building [50] or at their entrance [52]. Only two of these studies differentiated between indoor and outdoor stairs [50, 52]; a third study focused exclusively on outdoor stairs [51], while the remaining two studies did not include this information. All of these studies simply inquired about the presence or absence of stairs. None of these authors measured any structural features of stairs such as their dimensions, or the presence of handrails. In two studies, respondents were asked whether they considered stairs at the workplace to be safe, pleasant or accessible [48, 49]. Four of the five studies were undertaken exclusively in urban settings. Three of the five studies included a measure of terrain (slope and curved pathways).

**Table 3:** Summary of Articles That Assessed Stairs

Articles That Included Questions on Stairs (*n* = 5)	Age of Participants	Geographic Location & Terrain	Walkability Measure Used	Differentiated Between Indoor & Outdoor Stairs	Question (s) on Stairs
De Bourdeaudhuij et al. (2003) [48], De Bourdeaudhuij et al. (2005) [49]	2003: 18–65^a^ 2005: Sample 1: Mean = 35.1 (SD = 11.5); Sample 2: Mean = 34.1 (SD = 12.3)^b^	Urban (no information on terrain) Compared (city centre, suburbs, & countryside); no information on terrain	Self-created items on walkability	N (both papers)	“Are the stairs at your work accessible? Safe? Pleasant?” (Y, N, NA)
Kerr et al. (2011) [50]	66+	Urban; Assessed curved paths and path with moderate slope	Audit of Physical Activity Resources for Seniors (APARS)	Y	“Outside stairways (not from building)” (Y/N) “> 2 staircase” (Y/N), “> 1 staircase visible from main entrance” (Y/N)
Koh et al. (2015) [51]	65+	Urban; Assessed slopes in neighborhood	Self-created items on walkability	N (appears to focus on outdoor stairs only)	“There are few stairs/slopes in my neighbourhood” [“Strongly disagree” to “Strongly agree” (4-point scale)]
Tsai et al. (2013) [52]	75–81	Urban; Terrain was one variable they looked at (defined as hilly terrain and poor street conditions)	Self-created items on walkability	Y	Survey questions not written out in article but they asked about environmental mobility barriers which includes presence of outdoor or indoor stairs in entrances (Y/N)

^a^Note that older persons made up a small portion of this sample

^b^Age range was not specified in the article

Three of the four authors included their measures of stairs in the analysis of physical activity patterns. Composite variables, which included the items on stairs, were developed by Tsai et al. [52] and De Bourdeaudhuij et al. [48]. Tsai et al. [52] grouped the presence of indoor/outdoor stairs with other environmental barriers (no elevators, heavy doors, slippery floor, and inadequate lighting) to create a variable termed as “entrance”. Participants who reported an “entrance” barrier had a significantly higher odds of engaging in low or moderate amounts versus high amounts of walking for errands than those who reported no “entrance” barrier. A stratified analysis showed that this negative association was only significant among those who lived alone. De Bourdeaudhuij et al. [48] included items on stairs in a composite measure of the participants’ worksite environment, but the majority of their participants were not seniors. This worksite environment variable was significantly positively associated with vigorous physical activity in women and with walking in men. In their 2005 study, they compared environmental correlates of physical activity between Portuguese and Belgian adults. However, the worksite environment was not significantly correlated with physical activity in either group in this more recent study. In the Kerr et al. [50] study of 147 older persons, authors reported a significant positive correlation between the presence of outside stairways and amount of sedentary time.

## Discussion

This is the first review to describe the inclusion of stair measures in studies examining the relationship between walkability and physical activity. Given the omnipresence of stairs in outdoor environments, the challenges reported by older persons in safely navigating stairs [14, 53, 54] and the variability in stair design (particularly in the less regulated outdoor environment), it was surprising to see how infrequently authors reported any measures of stairs. While measures of grades or slopes, sidewalk curbs, uneven surfaces and ramps, were included in a number of walkability indices, the presence or absence of stairs and descriptions of stair features were absent from 97.6% of the primary studies. Variables to capture structural features of stairs such as handrail dimensions, tread width, stair height, or the number of stairs in a run, were entirely missing from this literature. These results are also intriguing because stairs have been identified as a specific environmental attribute that can be used to enhance the benefits of physical activity if an appropriate level of environmental press is achieved. That is, an older person’s environment poses enough challenges for them to maximize physical activity benefits, but not so much so that it leads them to incur negative outcomes [9].

None of the commonly used walkability scales integrated items on stairs. It may be that stairs have been excluded from these scales because authors have relied heavily on accessible data such as urban design data commonly used in geographic information systems rather than data used to assess smaller-scale characteristics [55]. Additionally, it can be argued that city planners are most concerned with sidewalks, curbs, cross-walks and other features of walking routes that are within their mandate to modify or control. Steps and stairs to the entrances of homes and buildings are more likely to be located on private rather than public property and therefore fall outside the responsibility of these public authorities. However, regardless of their location, steps and stairs that are part of a walking route must be taken into account if we are going to better understand the relationships between walking, physical activity and the built environment among older persons.

Our observation of an abundance of objective measures of walkability is consistent with the findings of Mackenbach et al. [56], who examined relationships between environmental factors and obesity. Only 17 of the 92 studies included in their review used a combination of objective and subjective measures. Given the known relationships between fear of falling and physical activity among older persons, a combination of subjective and objective measures is important to consider in developing items related to stair use.

A number of authors [13, 56–59] have called for standardized environmental measures given the challenges of making comparisons across studies when there is such substantial variability in measures used. We purport that researchers whose knowledge translation aims are to improve the walkability of neighbourhoods must include measures of stairs. An ad hoc approach to such measurement would be unhelpful. Comparable measures of stairs are needed to advance both research on walkability as well as to inform programs and policies that aim to improve these structures in our communities [60]. Design standards (e.g. U.S. ADA Stair and Handrail Design Specifications) and ergonomic studies that examine the relationships between structural features of stairs and kinematic measures [36, 61–63] provide an important starting point to identify the specific features of stairs that should be measured. There are also useful performance measures that assess actual ability to climb stairs (e.g. timed stair climb [64]); and behavioural measures that assess perceived ability to climb stairs (e.g. falls efficacy [65]). Augmenting walkability studies with these measures would help to further elucidate the relationships between walkability and physical activity.

Spatial heterogeneity in sampling physical attributes of the environment is a related, and thorny issue [27]. Pre-existing spatial boundaries have often been used to define sampling units but these boundaries do not necessarily demarcate preferred walking routes [55, 66]. There are new sampling strategies being proposed that could inform approaches for sampling stairs on walking routes. For instance, Milton et al. [66] describe a qualitative approach to define spatial units, noting that spatial units that are convenient for the purposes of sampling (e.g. administrative units, census tract enumeration areas etc.) may not reflect patterns of social interactions, service utilization or preferred walking routes among older persons. Their novel use of geographic information systems to map the actual walking routes of older persons, provides an interesting approach that could be applied to studies of walkability, physical activity and stairs; to identify which stairs are avoided and/or frequently traversed by older persons in comparison with younger persons in their neighbourhood. These might also be used to consistently document the slope or other features of the terrain, which may also affect walkability. Additionally, advances in creating navigational systems for those with visual impairments [67] that integrate geographic information systems data with information about visual landmarks captured by hand-held cameras, can be used to re-examine whether and how stairs feature in walking routes and subsequently, to guide sampling approaches.

The inclusion of stair measures in studies examining the relationships between walkability and physical activity, is arguably pertinent for all age groups, but especially true for older persons, and would yield evidence needed by planners who are trying to optimize the built environment for all age groups. Including descriptors of stairs in studies of walkability and the physical environment is also germane to the introduction of more substantial universal access standards and legislation in many jurisdictions [68–71]. Furthermore, an aging population, the desire of older persons to age at home, and government policies to support older persons’ independence and age-friendly communities, all provide an important impetus for work in this area.

The main limitation of this review is that we only looked at systematic reviews and primary studies examining the relationship between walkability and physical activity. The search terms “built environment” and “neighbourhood environment” were not used and may have yielded additional studies examining relationships between the outdoor physical environment and physical activity or obesity. Although we did not systematically identify reviews on these latter topics, we did a text search of 13 systematic reviews we have referred to in this manuscript that are in these other domains, and 6 reviews from a PubMed search that included the terms “built environment” and “neighbourhood environment”, and noted a similar deficit. Only three of these 19 reviews included any mention of stairs. Van Cauwenberg et al. [13] noted that in one of the primary studies they included, there was a negative relationship between the objectively measured presence of slopes and/or stairs and the utility of a street section for transportation walking. Gray [31] outlined the principles of universal design. One of those principles, flexibility in use, was defined as an option for use of ramps, stairs, escalators, elevators, and lifts. Haselwandter [72] described staircases as one feature of the built environment reporting that in one study they examined, older persons perceived the presence of outdoor stairs as a barrier to walking, with the inclusion of stair handrails as a facilitator for physical activity.

We did not extract information about the geography of the physical environment for primary studies (for example an urban, suburban or rural environment). While the presence of stairs and the terrain may vary in geographic locations, only five authors assessed stairs. Measures of stairs need to be added as a standard measure to walkability studies to allow an examination of interactions between stairs and geographic features of the environment in studies of walkability and physical activity among older persons.

## Conclusions

This scoping review demonstrates the lack of attention to an important feature of the outdoor built environment – stairs. Both structural (objective) and subjective (perception of user) measures of stairs need to be consistently used, and sampling strategies to select outdoor stairs for inclusion require development. These measures should be developed in consultation with diverse sectors that make planning and policy decisions influencing the built environment. Measures of walkability and sampling strategies for stairs must traverse the artificial public and private boundaries that fail to take into account the actual walking routes of older persons, which start from the doorstep of their residence. The addition of these measures would significantly augment the utility and comparability of studies examining relationships between walkability and physical activity.

## Additional file

Additional file 1: **Table S1.** Summary of Walkability Measures and Stair Data Extracted from Primary Studies (Grouped by Systematic Reviews). (DOCX 118 kb)

## Abbreviations

ANEWS: Abbreviated Neighborhood Environment Walkability Scale
APRARS: Audit of Physical Activity Resources for Seniors
GIS: Geographic Information Systems
IPAQ-E: International Physical Activity Questionnaire Environmental Module
NBOT: Neighborhood Brief Observation Tool
NEWS: Neighborhood Environment Walkability Scale
NOS: Neighborhood Open Space
NWQ-CS: Neighborhood Walking Questionnaire for Chinese Seniors
OPAL: Older Peoples Active Living
PRISMA: Preferred Reporting Items for Systematic Review and Meta-Analysis
SES: Socio-Economic Status
SPACES: Systematic Pedestrian and Cycling Environment Scan
UMBECS: University of Miami Built Environment Coding System
ZHTS: Zhongshan Household Travel Survey
