# Supplementary material for: Examining the relationships between walkability and physical activity among older persons: what about stairs?
Source: BMC Public Health. 2018 Aug 17;18:1025. doi: 10.1186/s12889-018-5945-0 (PMC6098658; doi:10.1186/s12889-018-5945-0)
Supplement: Supplementary file 1 — Table S1. Summary of Walkability Measures and Stair Data Extracted from Primary Studies (Grouped by Systematic Reviews). (DOCX 118 kb) [file 12889_2018_5945_MOESM1_ESM.docx]

**Table S1 Summary of Walkability Measures and Stair Data Extracted from Primary Studies (Grouped by Systematic Reviews)**

| **Author and Year of Review (Subcategory: Countries)** | **Number of Primary Articles** | **Names and Years of Primary Articles**  **(n = 324)^1^** | **Age of Participants** | **Measures Walkability?** | | **Name of Walkability Measure** | **Objective vs. Subjective Measure of Walkability^6^** | **Assesses Stairs?** | |
| --- | --- | --- | --- | --- | --- | --- | --- | --- | --- |
|  |  |  |  | **No** | **Yes** |  |  | **No** | **Yes** |
| **Barnett et al. (2017)** [1] **100** | | | | | | | | | |
| Australia | 13 | Aird & Buys (2015) [2] | 55+ |  | ✓ | Self-created items | Subjective | ✓ |  |
|  |  | Bird et al. (2009) [3] | 60+ |  | ✓ | Neighborhood Environment Walkability Scale (NEWS) | Subjective | ✓ |  |
|  |  | Bird et al. (2010) [4] | 60+ |  | ✓ | Neighborhood Environment Walkability Scale (NEWS) | Subjective | ✓ |  |
|  |  | Cerin et al. (2016) [5] | 60+ |  | ✓ | Geographic Information Systems (GIS) | Objective | ✓ |  |
|  |  | Espinel et al. (2015) [6] | 65+ | ✓ |  |  |  |  |  |
|  |  | Lim & Taylor (2005) [7] | 65+ | ✓ |  |  |  |  |  |
|  |  | MacNiven et al. (2014) [8] | 65+ | ✓ |  |  |  |  |  |
|  |  | Merom et al. (2015)[9] | 65+ |  | ✓ | Neighborhood Environment Walkability Scale (NEWS) | Subjective | ✓ |  |
|  |  | Nathan et al. (2012) [10] | 65-84 |  | ✓ | Geographic Information Systems (GIS) | Objective | ✓ |  |
|  |  | Nathan et al. (2014a) [11] | 53-94 |  | ✓ | Abbreviated Neighborhood Environment Walkability Scale (ANEWS) | Subjective | ✓ |  |
|  |  | Nathan et al. (2014b) [12] | 53-94 |  | ✓ | Geographic Information Systems (GIS) and Neighborhood Environment Walkability Scale (NEWS) | Objective | ✓ |  |
|  |  | Nathan et al. (2014c) [13] | 53-94 |  | ✓ | Geographic Information Systems (GIS) | Objective | ✓ |  |
|  |  | Villanueva et al. (2014) [14] | 18+ |  | ✓ | Geographic Information Systems (GIS) | Objective | ✓ |  |
| Belgium | 2 | Van Cauwenberg et al. (2016) [15] | 65+ |  | ✓ | Geographic Information Systems (GIS) | Objective | ✓ |  |
|  |  | Van Holle et al. (2016) [16] | 65+ |  | ✓ | Neighborhood Environment Walkability Scale (NEWS) | Subjective | ✓ |  |
| Brazil | 1 | Salvador et al. (2010) [17] | 60+ |  | ✓ | Neighborhood Environment Walkability Scale (NEWS) | Subjective | ✓ |  |
| Canada | 10 | Chad et al. (2005) [18] | 50+ | ✓ |  |  |  |  |  |
|  |  | Chaudhury et al. (2016) [19] | 60+ |  | ✓ | Neighborhood Environment Walkability Scale (NEWS) | Subjective | ✓ |  |
|  |  | De Melo (2013) [20] | 62+ |  | ✓ | Neighborhood Environment Walkability Scale (NEWS) | Subjective | ✓ |  |
|  |  | De Melo et al. (2010) [21] | 65+ |  | ✓ | Neighborhood Environment Walkability Scale (NEWS) | Subjective | ✓ |  |
|  |  | Gauvin et al. (2012) [22] | 67-84 |  | ✓ | Geographic Information Systems (GIS) & self-created items | Both | ✓ |  |
|  |  | Hirsch et al. (2016) [23] | 65+ |  | ✓ | (Street Smart) Walk Score | Objective | ✓ |  |
|  |  | Julien et al. (2015) [24] | 68-84 | ✓ |  |  |  |  |  |
|  |  | Pan et al. (2009) [25] | 15+ |  | ✓ | Self-created items | Subjective | ✓ |  |
|  |  | Spinney & Milward (2014) [26] | 65+ | ✓ |  |  |  |  |  |
|  |  | Winters et al. (2015) [27] | 65+ |  | ✓ | (Street Smart) Walk Score | Objective | ✓ |  |
| China | 1 | Zhang et al. (2014) [28] | 60+ |  | ✓ | Zhongshan Household Travel Survey (ZHTS) | Subjective | NA^4^ |  |
| Colombia | 1 | Gómez et al. (2010) [29] | 60+ |  | ✓ | Geographic Information Systems (GIS) | Objective | ✓ |  |
| Czech Republic, Poland, & Slovakia (pooled analysis) | 1 | Pelclová et al. (2012) [30] | 60+ |  | ✓ | Abbreviated Neighborhood Environment Walkability Scale (ANEWS) | Subjective | ✓ |  |
| Hong Kong | 2 | Cerin et al. (2016) [31] | 65+ |  | ✓ | Geographic Information Systems (GIS) | Objective | ✓ |  |
|  |  | Cerin et al. (2013) [32] | 65+ |  | ✓ | Neighborhood Walking Questionnaire for Chinese Seniors (NWQ-CS) | Subjective | ✓ |  |
| Iceland | 1 | Arnadottir et al. (2009) [33] | 65+ | ✓ |  |  |  | ✓ |  |
| Iran | 1 | Lotfi & Koohsari (2011) [34] | 65+ |  | ✓ | Geographic Information Systems (GIS) | Objective | ✓ |  |
| Ireland | 2 | McKee et al. (2015) [35] | 65+ | NA^2^ |  |  |  |  |  |
|  |  | Murtagh et al. (2015) [36] | 60+ | ✓ |  |  |  |  |  |
| Japan | 7 | Carvalho Sampaio et al. (2012) [37] | 65+ | ✓ |  |  |  |  |  |
|  |  | Chen et al. (2013) [38] | 20-79 |  | ✓ | Neighborhood Environment Walkability Scale (NEWS) | Subjective | ✓ |  |
|  |  | Hanibuchi et al. (2011) [39] | 65+ |  | ✓ | Geographic Information Systems (GIS) | Objective | ✓ |  |
|  |  | Inoue et al. (2011) [40] | 65-74 |  | ✓ | International Physical Activity Questionnaire Environmental Module (IPAQ-E) | Subjective | ✓ |  |
|  |  | Sewo Sampaio et al. (2013) [41] | 60+ | ✓ |  |  |  |  |  |
|  |  | Tanaka et al. (2016) [42] | 65+ | ✓ |  |  |  |  |  |
|  |  | Tsunoda et al. (2012) [43] | 65-85 |  | ✓ | International Physical Activity Questionnaire Environmental Module (IPAQ-E) | Subjective | ✓ |  |
| Lithuania | 1 | Baceviciene & Alisauskas (2013) [44] | 60-89 | ✓ |  |  |  |  |  |
| Malaysia | 1 | Kaur et al. (2015) [45] | 60+ | ✓ |  |  |  |  |  |
| Netherlands | 4 | Bocker et al. (2006) [46] | 65+ | ✓ |  |  |  |  |  |
|  |  | Jongeneel-Grimen et al. (2013) [47] | 18-84 | ✓ |  |  |  |  |  |
|  |  | Jongeneel-Grimen et al. (2014) [48] | 18-84 | ✓ |  |  |  |  |  |
|  |  | Timmermans et al. (2016) [49] | 66-85 |  | ✓ | Geographic Information Systems (GIS) | Objective | ✓ |  |
| Norway | 1 | Piro et al. (2006) [50] | 75/76 | ✓ |  |  |  |  |  |
| Singapore | 1 | Koh et al. (2015) [51] | 65+ |  | ✓ | Self-created items | Subjective |  | ✓ |
| South Africa | 1 | Kolbe-Alexander et al. (2015) [52] | 50+ |  | ✓ | Neighborhood Environment Walkability Scale (NEWS) | Subjective | ✓ |  |
| South Korea | 2 | Lee et al. (2015) [53] | NA^3^ |  |  |  |  |  |  |
|  |  | Yeom et al. (2011) [54] | 65+ | ✓ |  |  |  |  |  |
| Thailand | 1 | Asawachaisuwikrom (2001) [55] | 60+ |  | ✓ | Neighborhood Environment Walkability Scale (NEWS) | Subjective | ✓ |  |
| United Kingdom | 10 | Davis et al. (2011) [56] | 70+ | ✓ |  |  |  |  |  |
|  |  | Fox et al. (2011) [57] | 70+ |  | ✓ | Older Peoples Active Living (OPAL) questionnaire | Subjective | NA^4^ |  |
|  |  | Jefferis et al. (2014) [58] | 70-93 |  | ✓ | Self-created questions | Subjective | ✓ |  |
|  |  | McMurdo et al. (2012) [59] | 65+ |  | ✓ | Older Peoples Active Living (OPAL) questionnaire | Subjective | NA^4^ |  |
|  |  | Persson & While (2012) [60] | 60+ |  | ✓ | Self-created questions | Subjective | NA^4^ |  |
|  |  | Sugiyama & Thompson (2007) [61] | 65+ |  | ✓ | Neighborhood Open Space (NOS) | Subjective | ✓ |  |
|  |  | Sugiyama et al. (2009) [62] | 65+ |  | ✓ | Neighborhood Open Space (NOS) | Subjective | ✓ |  |
|  |  | Sniehotta et al. (2013) [63] | 65+ |  | ✓ | Older Peoples Active Living (OPAL) questionnaire | Subjective | NA^4^ |  |
|  |  | Thompson et al. (2012) [64] | 65+ |  | ✓ | Neighborhood Open Space (NOS) | Subjective | ✓ |  |
|  |  | Thompson et al. (2011) [65] | 70+ |  | ✓ | Questions adapted from the Neighborhood Environment Walkability Scale (NEWS) | Subjective | ✓ |  |
| United States | 36 | Bracy et al. (2014) [66] | 20+ |  | ✓ | Geographic Information Systems (GIS) | Objective | ✓ |  |
|  |  | Cain et al. (2014) [67] | 6+ |  | ✓ | Geographic Information Systems (GIS) | Objective | ✓ |  |
|  |  | Carlson et al. (2014)[68] | 66+ |  | ✓ | Geographic Information Systems (GIS) & Neighborhood Environment Walkability Scale (NEWS) | Both | ✓ |  |
|  |  | Carlson et al. (2012) [69] | 65+ |  | ✓ | Geographic Information Systems (GIS) & Neighborhood Environment Walkability Scale (NEWS) | Both | ✓ |  |
|  |  | Ding et al. (2014) [70] | 66+ |  | ✓ | Geographic Information Systems (GIS) & Neighborhood Environment Walkability Scale (NEWS) | Both | ✓ |  |
|  |  | Frank et al. (2010) [71] | 65+ |  | ✓ | Self-created items | Objective | ✓ |  |
|  |  | Gallagher et al. (2012) [72] | 60+ |  | ✓ | Neighborhood Environment Walkability Scale (NEWS) | Subjective | ✓ |  |
|  |  | Grant-Savela (2010) [73] | 60+ | ✓ |  |  |  |  |  |
|  |  | Hall & McAuley (2010) [74] | 59-84 |  | ✓ | Geographic Information Systems (GIS) & Neighborhood Environment Walkability Scale (NEWS) | Both | ✓ |  |
|  |  | Han et al. (2016) [75] | 45-75 | ✓ |  |  |  |  |  |
|  |  | Hannon et al. (2012) [76] | 65+ |  | ✓ | Self-created items | Subjective | ✓ |  |
|  |  | James et al. (2013) [77] | 25-55 | ✓ |  |  |  |  |  |
|  |  | Kerr et al. (2011) [78] | 66+ |  | ✓ | Audit of Physical Activity Resources for Seniors (APARS) | Objective |  | ✓ |
|  |  | King et al. (2006) [79] | 18-85 |  | ✓ | Neighborhood Environment Walkability Scale (NEWS) | Subjective | ✓ |  |
|  |  | King et al. (2003) [80] | 50-65 |  | ✓ | Self-created items | Subjective | ✓ |  |
|  |  | Latham & Williams (2015) [81] | 50+ | ✓ |  |  |  |  |  |
|  |  | Lee et al. (2009) [82] | Mean = 70 (SD = 6.8) | ✓ |  |  |  |  |  |
|  |  | Li et al. (2005a) [83] | 65+ |  | ✓ | Self-created items | Subjective | ✓ |  |
|  |  | Li et al. (2005b) [84] | 65+ |  | ✓ | Geographic Information Systems (GIS) | Objective | ✓ |  |
|  |  | Li et al. (2015) [85] | 55+ | ✓ |  |  |  |  |  |
|  |  | Maisel (2006) [86] | 65+ |  | ✓ | Neighborhood Environment Walkability Scale (NEWS) | Subjective | ✓ |  |
|  |  | Leon et al. (2009) [87] | 65+ | ✓ |  |  |  |  |  |
|  |  | Morris et al. (2008) [88] | Sample 1: 59-84; Sample 2: Mean = 46.1 (SD = 9.5) |  | ✓ | Neighborhood Environment Walkability Scale (NEWS) | Subjective | ✓ |  |
|  |  | Michael et al. (2006) [89] | 65+ |  | ✓ | Geographic Information Systems (GIS) & self-created items | Both | ✓ |  |
|  |  | Michael et al. (2010) [90] | 65+ | ✓ |  |  |  |  |  |
|  |  | Mowen et al. (2007) [91] | 50+ | ✓ |  |  |  | ✓ |  |
|  |  | Nagel et al. (2008) [92] | 65+ |  | ✓ | Geographic Information Systems (GIS) | Objective | ✓ |  |
|  |  | Perry et al. (2013) [93] | 50-79 |  | ✓ | Geographic Information Systems (GIS) | Objective | ✓ |  |
|  |  | Procter-Gray (2015) [94] | 70+ |  | ✓ | Geographic Information Systems (GIS) | Objective | ✓ |  |
|  |  | Satariano et al. (2010) [95] | 65+ |  | ✓ | Geographic Information Systems (GIS) & Neighborhood Environment Walkability Scale (NEWS) | Both | ✓ |  |
|  |  | Shores et al. (2009) [96] | 65+ |  | ✓ | Neighborhood Environment Walkability Scale (NEWS) | Subjective | ✓ |  |
|  |  | Shin et al. (2011) [97] | 55-84 |  | ✓ | Geographic Information Systems (GIS) | Objective | ✓ |  |
|  |  | Towne et al. (2016)[98] | 50+ |  | ✓ | (Street Smart) Walk Score | Objective | ✓ |  |
|  |  | Troped et al. (2014)[99] | 57-85 |  | ✓ | Geographic Information Systems (GIS) | Objective | ✓ |  |
|  |  | Wang & Lee (2010) [100] | 65+ |  | ✓ | Geographic Information Systems (GIS) & self-created items | Both | ✓ |  |
|  |  | Wilcox et al. (2003) [101] | 50+ |  | ✓ | Self-created items |  | ✓ |  |
| **Cerin et al. (2017) 42** [102] | | | | | | | | | |
| Australia | 5 | Boruff et al. (2012) [103] | Mean = 76.82 (SD = 7.43) |  | ✓ | Geographic Information Systems (GIS) | Objective | ✓ |  |
|  |  | Garrard (2013) [104] | 60+ |  | ✓ | Self-created items | Subjective | ✓ |  |
|  |  | Nathan et al. (2014a) [11] | 53-94 |  | ✓ | Neighborhood Environment Walkability Scale (NEWS) | Subjective | ✓ |  |
|  |  | Nathan et al. (2014b) [12] | 53-94 |  | ✓ | Neighborhood Environment Walkability Scale (NEWS) | Subjective | ✓ |  |
|  |  | Nathan et al. (2014c) [13] | 53-94 |  | ✓ | Geographic Information Systems (GIS) and Neighborhood Environment Walkability Scale (NEWS) | Objective | ✓ |  |
| Belgium | 4 | Van Cauwenberg et al. (2012) [105] | 60+ |  | ✓ | Geographic Information Systems (GIS) & self-created items | Objective | ✓ |  |
|  |  | Van Cauwenberg et al. (2016) [15] | 65+ |  | ✓ | Geographic Information Systems (GIS) | Objective | ✓ |  |
|  |  | Van Holle et al. (2014) [106] | 65+ |  | ✓ | Geographic Information Systems (GIS) | Objective | ✓ |  |
|  |  | Van Holle et al. (2016) [16] | 65+ |  | ✓ | Neighborhood Environment Walkability Scale (NEWS) | Subjective | ✓ |  |
| Brazil | 3 | Corseuil Giehl et al. (2011) [107] | 60+ |  | ✓ | Neighborhood Environment Walkability Scale (NEWS) | Subjective | ✓ |  |
|  |  | Corseuil Giehl et al. (2016)[108] | 60+ |  | ✓ | Geographic Information Systems (GIS) | Objective | ✓ |  |
|  |  | Corseuil Giehl et al. (2017) [109] | 60+ |  | ✓ | Neighborhood Environment Walkability Scale (NEWS) | Subjective | ✓ |  |
| Canada | 4 | Barnes et al. (2016) [110] | 45+ |  | ✓ | (Street Smart) Walk Score | Objective | ✓ |  |
|  |  | Chudyk et al. (2015) [111] | 60+ |  | ✓ | (Street Smart) Walk Score | Objective | ✓ |  |
|  |  | Moniruzzaman et al. (2013) [112] | 55+ |  | ✓ | Self-created items | Objective | ✓ |  |
|  |  | Moniruzzaman et al. (2015) [113] | 55+ |  | ✓ | Self-created items | Objective | ✓ |  |
| Czech Republic, Poland, & Slovakia (pooled analysis) | 1 | Pelclová et al. (2012) [30] | 60+ |  | ✓ | Neighborhood Environment Walkability Scale (NEWS) | Subjective | ✓ |  |
| Finland | 1 | Tsai et al. (2013) [114] | 75-81 |  | ✓ | Self-created items | Subjective |  | ✓ |
| Hong Kong | 4 | Barnett et al. (2016) [115] | 65+ |  | ✓ | Neighborhood Environment Walkability Scale (NEWS) & Neighborhood Walking Questionnaire for Chinese Seniors (NWQ-CS) | Subjective | ✓ |  |
|  |  | Cerin et al. (2013a) [116] | 65+ |  | ✓ | Neighborhood Environment Walkability Scale (NEWS) | Subjective | ✓ |  |
|  |  | Cerin et al. (2013b) [117] | 65+ |  | ✓ | Neighborhood Walking Questionnaire for Chinese Seniors (NWQ-CS) | Subjective | ✓ |  |
|  |  | Cerin et al. (2014) [118] | 65+ |  | ✓ | Neighborhood Environment Walkability Scale (NEWS) & Neighborhood Walking Questionnaire for Chinese Seniors (NWQ-CS) | Subjective | ✓ |  |
| Japan | 1 | Inoue et al. (2011) [40] | 65-74 |  | ✓ | International Physical Activity Questionnaire Environmental Module (IPAQ-E) | Subjective | ✓ |  |
| Netherlands | 1 | Etman et al. (2014) [119] | 65+ |  | ✓ | Geographic Information Systems (Google Maps) | Objective | ✓ |  |
| Singapore | 1 | Nyunt et al. (2015) [120] | 55+ |  | ✓ | Geographic Information Systems (GIS) & Neighborhood Environment Walkability Scale (NEWS) | Both | ✓ |  |
| South Africa | 1 | Kolbe-Alexander et al. (2015) [52] | 50+ |  | ✓ | Geographic Information Systems (GIS) | Objective | ✓ |  |
| United Kingdom | 2 | Davis et al. (2011) [56] | 70+ | ✓ |  |  |  |  |  |
|  |  | Sugiyama & Thompson (2007) [61] | 65+ |  | ✓ | Neighborhood Open Space (NOS) | Subjective | ✓ |  |
| United States | 14 | Bracy et al. (2014) [66] | 20+ |  | ✓ | Geographic Information Systems (GIS) | Objective | ✓ |  |
|  |  | Cain et al. (2014) [67] | 6+ |  | ✓ | Geographic Information Systems (GIS) | Objective | ✓ |  |
|  |  | Carlson et al. (2012) [69] | 65+ |  | ✓ | Geographic Information Systems (GIS) & Neighborhood Environment Walkability Scale (NEWS) | Both | ✓ |  |
|  |  | Ding et al. (2014) [70] | 66+ |  | ✓ | Geographic Information Systems (GIS) & Neighborhood Environment Walkability Scale (NEWS) | Both | ✓ |  |
|  |  | Frank et al. (2010) [71] | 65+ |  | ✓ | Self-created items | Objective | ✓ |  |
|  |  | King (2008) [121] | 65+ |  | ✓ | Systematic Pedestrian and Cycling Environment Scan (SPACES) & Neighborhood Brief Observation Tool (NBOT) | Objective | ✓ |  |
|  |  | King et al. (2006) [79] | 18-85 |  | ✓ | Neighborhood Environment Walkability Scale (NEWS) | Subjective | ✓ |  |
|  |  | King et al. (2011) [122] | 66+ | ✓ |  |  |  |  |  |
|  |  | Maisel (2016) [86] | 65+ |  | ✓ | Neighborhood Environment Walkability Scale (NEWS) | Subjective | ✓ |  |
|  |  | Leon et al. (2009) [87] | 65+ | ✓ |  |  |  |  |  |
|  |  | Mitchell (2012) [123] | 65+ |  | ✓ | Neighborhood Environment Walkability Scale (NEWS) | Subjective | ✓ |  |
|  |  | Patterson & Chapman (2004) [124] | 70+ |  | ✓ | New Urbanism Index & Neighborhood Resident Survey | Subjective | ✓ |  |
|  |  | Procter-Grey et al. (2015) [94] | 70+ |  | ✓ | Geographic Information Systems (GIS) & self-created items |  | ✓ |  |
|  |  | Shigematsu et al. (2009) [125] | 20+ |  | ✓ | Neighborhood Environment Walkability Scale (NEWS) | Subjective | ✓ |  |
| **Grasser et al. (2013) 34** [126] | | | | | | | | | |
| Australia | 3 | Cerin et al. (2007)[127] | 20-65 |  | ✓ | Geographic Information Systems (GIS) & Neighborhood Environment Walkability Scale (NEWS) | Both | ✓ |  |
|  |  | Owen et al. (2007) [128] | 20-65 |  | ✓ | Geographic Information Systems (GIS) | Objective | ✓ |  |
|  |  | Owen et al. (2010) [129] | 18-65 |  | ✓ | Geographic Information Systems (GIS) & Neighborhood Environment Walkability Scale (NEWS) | Both | ✓ |  |
| Belgium | 2 | Van Dyck et al. (2010a) [130] | 20-65 |  | ✓ | Geographic Information Systems (GIS) | Objective | ✓ |  |
|  |  | Van Dyck et al. (2010b) [131] | 20-65 |  | ✓ | Geographic Information Systems (GIS) | Objective | ✓ |  |
| Canada | 1 | Pouliou & Elliott (2010) [132] | 20+ |  | ✓ | Geographic Information Systems (GIS) | Objective | ✓ |  |
| United Kingdom | 1 | Coombes et al. (2010) [133] | 16+ |  | ✓ | Geographic Information Systems (GIS) | Objective | ✓ |  |
| United States | 27 | Berke et al. (2007) [134] | 65+ |  | ✓ | Geographic Information Systems (GIS) | Objective | ✓ |  |
|  |  | Bodea et al. (2008) [135] | 21+ |  | ✓ | Geographic Information Systems (GIS) | Objective | ✓ |  |
|  |  | Boer et al. (2007) [136] | 5+ |  | ✓ | Geographic Information Systems (GIS) | Objective | ✓ |  |
|  |  | Brown et al. (2009) [137] | 25-64 |  | ✓ | Geographic Information Systems (GIS) | Objective | ✓ |  |
|  |  | Chatman (2009) [138] | Mean: 46.16 (SD: 17.04) |  | ✓ | Geographic Information Systems (GIS) | Objective | ✓ |  |
|  |  | Forsyth et al. (2007) [139] | Mean: 47.04 (Median: 45) |  | ✓ | Geographic Information Systems (GIS) | Objective | ✓ |  |
|  |  | Forsyth et al. (2008) [140] | Mean: 47.04 (Median: 45) |  | ✓ | Geographic Information Systems (GIS) | Objective | ✓ |  |
|  |  | Forsyth et al. (2009) [141] | Mean: 47.04 (Median: 45) |  | ✓ | Geographic Information Systems (GIS) | Objective | ✓ |  |
|  |  | Frank et al. (2004) [142] | 16-95 |  | ✓ | Geographic Information Systems (GIS) | Objective | ✓ |  |
|  |  | Frank et al. (2006) [143] | 20-65 |  | ✓ | Geographic Information Systems (GIS) | Objective | ✓ |  |
|  |  | Frank et al. (2007) [144] | 16-92 |  | ✓ | Geographic Information Systems (GIS) | Objective | ✓ |  |
|  |  | Frank et al. (2008) [145] | 25+ |  | ✓ | Geographic Information Systems (GIS) | Objective | ✓ |  |
|  |  | Frank et al. (2009) [146] | 25-65 |  | ✓ | Geographic Information Systems (GIS) | Objective | ✓ |  |
|  |  | Huang et al. (2009) [147] | 18+ |  | ✓ | Geographic Information Systems (GIS) | Objective | ✓ |  |
|  |  | Kitamura et al. (1997) [148] | 16+ |  | ✓ | Geographic Information Systems (GIS) and self-created items | Both | ✓ |  |
|  |  | Lee & Moudon (2006) [149] | 18+ |  | ✓ | Geographic Information Systems (GIS) and self-created items | Both | ✓ |  |
|  |  | Li et al. (2008) [150] | 50-75 |  | ✓ | Geographic Information Systems (GIS) | Objective | ✓ |  |
|  |  | Li et al. (2009) [151] | 50-75 |  | ✓ | Geographic Information Systems (GIS) | Objective | ✓ |  |
|  |  | Lopez (2007) [152] | Adults (specific ages unknown) |  | ✓ | Geographic Information Systems (GIS) | Objective | ✓ |  |
|  |  | McGinn et al. (2007) [153] | Adults (specific ages unknown) |  | ✓ | Geographic Information Systems (GIS) and self-created items | Both | ✓ |  |
|  |  | Oakes et al. (2007) [154] | 25+ |  | ✓ | Geographic Information Systems (GIS) | Objective | ✓ |  |
|  |  | Rundle et al. (2007) [155] | 30+ |  | ✓ | Geographic Information Systems (GIS) | Objective | ✓ |  |
|  |  | Rundle et al. (2009) [156] | Adults [Mean: 46.21 +/- 10.55 (SD); Median: 45] |  | ✓ | Geographic Information Systems (GIS) | Objective | ✓ |  |
|  |  | Sallis et al. (2009) [157] | 20-65 |  | ✓ | Walkability index (Geographic Information Systems) | Objective | ✓ |  |
|  |  | Scott et al. (2009) [158] | Adults |  | ✓ | Geographic Information Systems (GIS) | Objective | ✓ |  |
|  |  | Smith et al. (2008) [159] | 25-64 |  | ✓ | Geographic Information Systems (GIS) | Objective | ✓ |  |
|  |  | Zick et al. (2009) [160] | 25-64 |  | ✓ | Geographic Information Systems (GIS) | Objective | ✓ |  |
| **McCormack & Shiell (2011) 33** [161] | | | | | | | | | |
| Australia | 1 | Owen et al. (2007) [128] | 20-65 |  | ✓ | Geographic Information Systems (GIS) | Objective | ✓ |  |
| Canada | 1 | MacBeth (1999) [162] | NA^5^ | ✓ |  |  |  |  |  |
| Netherlands | 1 | Meurs & Haijer (2001) [163] | NA^5^ |  | ✓ | Self-created items | Objective | ✓ |  |
| United Kingdom | 1 | Painter (1996) [164] | NA^5^ | ✓ |  |  |  |  |  |
| United States | 29 | Bagley & Mokhtarian (2002) [165] | 16+ |  |  | Self-created items | Subjective | ✓ |  |
|  |  | Boone-Heinonen et al. (2010) [166] | 11-26 |  | ✓ | Geographic Information Systems (GIS) | Objective | ✓ |  |
|  |  | Boarnet et al. (2008) [167] | 5+ | ✓ |  |  |  |  |  |
|  |  | Brown & Werner (2008) [168] | Mean = 41 (SD: 13.82) | ✓ |  |  |  |  |  |
|  |  | Brown & Werner (2007) [169] | Mean = 41 (SD: 13.82) | ✓ |  |  |  |  |  |
|  |  | Burbridge & Goulias (2009) [170] | 5+ | ✓ |  |  |  |  |  |
|  |  | Cao et al. (2006) [171] | Median ages: 43.3, 43.8, 44, 46.5, 42, 44.3 |  | ✓ | Self-created items | Subjective | ✓ |  |
|  |  | Cao et al. (2009a) [172] | Mean ages of samples: 43.3, 47, 51.3, 43.4, 47.1, 54.7, 53.2, 45.6 |  | ✓ | Geographic Information Systems (GIS) & self-created items | Both | ✓ |  |
|  |  | Cao et al. (2009b) [173] | Mean ages of samples: 43.3, 47, 51.3, 43.4, 47.1, 54.7, 53.2, 45.6 |  | ✓ | Self-created items | Subjective | ✓ |  |
|  |  | Cao (2010) [174] | Mean ages of samples: 43.3, 47, 51.3, 43.4, 47.1, 54.7, 53.2, 45.6 |  | ✓ | Self-created items | Subjective | ✓ |  |
|  |  | Cao et al. (2007) [175] | Mean ages of samples: 36.6, 39, 41.2, 35.6, 38.4, 49.4, 42.7, 39.4 |  | ✓ | Self-created items | Both | ✓ |  |
|  |  | Chatman (2009) [138] | Mean: 46.16 (SD: 17.04) |  | ✓ | Geographic Information Systems (GIS) | Objective | ✓ |  |
|  |  | Cohen et al. (2009) [176] | Median ages of sample at baseline: 36.5, 38.5; Median ages of samples at follow-up: 37.1, 40.5 | ✓ |  |  |  |  |  |
|  |  | Coogan et al. (2009) [177] | 21-69 |  | ✓ | Geographic Information Systems (GIS) | Objective | ✓ |  |
|  |  | Evenson et al. (2005) [178] | 18+ |  | ✓ | Geographic Information Systems (GIS) & self-created items | Both | ✓ |  |
|  |  | Fitzhugh et al. (2010) [179] | Median ages of groups: 30 and 39.5 (included children & adolescents) | ✓ |  |  |  |  |  |
|  |  | Frank et al. (2007) [144] | 16-92 |  | ✓ | Geographic Information Systems (GIS) | Objective | ✓ |  |
|  |  | Greenwald & Boarnet (2001) [180] | 5+ | ✓ |  |  |  |  |  |
|  |  | Handy et al. (2008) [181] | Mean ages of samples: 43.3, 47, 51.3, 43.4, 47.1, 54.7, 53.2, 45.6 |  | ✓ | Geographic Information Systems (GIS) & self-created items | Both | ✓ |  |
|  |  | Handy et al. (2006) [182] | Mean ages of samples: 43.3, 47, 51.3, 43.4, 47.1, 54.7, 53.2, 45.6 |  | ✓ | Geographic Information Systems (GIS) & self-created items | Both | ✓ |  |
|  |  | Joh et al. (2008) check year [183] | 25+ and includes participants < this but unsure if it’s adults only or children as well |  | ✓ | Self-created items | Subjective | ✓ |  |
|  |  | Khattak & Rodriguez (2005) [184] | Median ages of 2 groups: 45.9 & 47.3 |  | ✓ | Geographic Information Systems (GIS) | Objective | ✓ |  |
|  |  | Lee et al. (2009) [82] | Mean = 70 (SD = 6.8) | ✓ |  |  |  |  |  |
|  |  | MacDonald et al. (2010) [185] | Mean = 52.1 (SD = 16.3) |  | ✓ | Geographic Information Systems (GIS) & self-created items | Both | ✓ |  |
|  |  | Pinjari et al. (2009)[186] | Mean: 44.8 |  | ✓ | Geographic Information Systems (GIS) | Objective | ✓ |  |
|  |  | Sallis et al. (2009) [157] | 20-65 |  | ✓ | Walkability index (Geographic Information Systems) | Objective | ✓ |  |
|  |  | Schwanen & Mokhtarian (2005) [187] | 18+ |  | ✓ | Self-created items | Subjective | ✓ |  |
|  |  | Shay et al. (2006) [188] | 16+ | ✓ |  |  |  |  |  |
|  |  | Wells & Yang (2008) [189] | 23-66 |  | ✓ | Geographic Information Systems (GIS) | Objective | ✓ |  |
| **Renalds et al. (2010) 19** [190] | | | | | | | | | |
| Australia | 1 | Wood et al. (2008) [191] | 18+ |  | ✓ | Geographic Information Systems (GIS) | Objective | ✓ |  |
| Austria | 1 | Titze et al. (2008) [192] | 15-60 | ✓ |  |  |  |  |  |
| Ireland | 1 | Leyden (2003) [193] | 18+ |  | ✓ | Self-created walkability index | Subjective | ✓ |  |
| Netherlands | 1 | Dijkstra et al. (2008) [194] | Mean: 21 (SD: 2.2) | ✓ |  |  |  |  |  |
| United Kingdom | 1 | Araya et al. (2006) [195] | 16-75 | ✓ |  |  |  |  |  |
| United States | 19 | Abildso et al. (2007) [196] | 18+ |  | ✓ | Self-created items | Subjective | ✓ |  |
|  |  | Berke et al. (2007) [134] | 65+ |  | ✓ | Geographic Information Systems (GIS) | Objective | ✓ |  |
|  |  | Bernstein et al. (2007) [197] | 18+ | ✓ |  |  |  |  |  |
|  |  | Brown et al. (2008) [198] | 70-100 |  | ✓ | University of Miami Built Environment Coding System (UMBECS) | Objective | ✓ |  |
|  |  | Clarke et al. (2008) [199] | 45+ | ✓ |  |  |  |  |  |
|  |  | Clarke et al. (2005) [200] | 65+ |  | ✓ | Geographic Information Systems (GIS) | Objective | ✓ |  |
|  |  | Cohen et al. (2008) [201] | 18+ |  | ✓ | Geographic Information Systems (GIS) | Objective | ✓ |  |
|  |  | Galea et al. (2005) [202] | 18+ | ✓ |  |  |  |  |  |
|  |  | Gordon-Larsen (2006) [203] | Adolescents (Grades 7-12) | ✓ |  |  |  |  |  |
|  |  | Grafova (2008) [204] | 5-18 |  | ✓ | Geographic Information Systems (GIS) | Objective | ✓ |  |
|  |  | Heinrich et al. (2008) [205] | 18-93 | ✓ |  |  |  |  |  |
|  |  | Lee et al. (2008) [206] | Elementary School Children |  | ✓ | Geographic Information Systems (GIS) | Objective | ✓ |  |
|  |  | Li et al. (2005) [84] | 65+ |  | ✓ | Geographic Information Systems (GIS) | Objective | ✓ |  |
|  |  | Li et al. (2008) [150] | 50-75 |  | ✓ | Geographic Information Systems (GIS) | Objective | ✓ |  |
|  |  | Lopez-Zetina et al. (2008) [207] | 16+ | ✓ |  |  |  |  |  |
|  |  | Nagel et al. (2008) [92] | 65+ |  | ✓ | Geographic Information Systems (GIS) | Objective | ✓ |  |
|  |  | Rundle et al. (2007) [155] | 30+ |  | ✓ | Geographic Information Systems (GIS) | Objective | ✓ |  |
|  |  | Wang et al. (2004) [208] | NA^5^ | ✓ |  |  |  |  |  |
| **Van Holle (2012) 69** [209] | | | | | | | | | |
| Austria | 3 | Stronegger et al. (2010) [210] | 15-60 | ✓ |  |  |  |  |  |
|  |  | Titze et al. (2007) [211] | University students (Mean: 23.8, SD: 4.1) | ✓ |  |  |  |  |  |
|  |  | Titze et al. (2008) [192] | 15-60 | ✓ |  |  |  |  |  |
| Belgium | 13 | De Bourdeaudhuij et al. (2003) [212] | 18-65 |  | ✓ | Self-created items | Subjective |  | ✓ |
|  |  | De Bourdeaudhuij et al. (2005) [213] | Sample 1: Mean = 35.1 (SD = 11.5); Sample 2: Mean = 34.1 (SD = 12.3) |  | ✓ | Self-created items | Subjective |  | ✓ |
|  |  | de Geus et al. (2005) [214] | 18-65 | ✓ |  |  |  |  |  |
|  |  | Owen et al. (2010) [129] | 18-65 |  | ✓ | Geographic Information Systems (GIS) & Neighborhood Environment Walkability Scale (NEWS) | Both | ✓ |  |
|  |  | Van Dyck et al. (2009) [215] | 20-65 |  | ✓ | Scale created items | Objective | ✓ |  |
|  |  | Van Dyck et al. (2010) [131] | 20-65 |  | ✓ | Geographic Information Systems (GIS) | Objective | ✓ |  |
|  |  | Van Dyck et al. (2011a) [216] | 20-65 |  | ✓ | Geographic Information Systems (GIS) | Objective | ✓ |  |
|  |  | Van Dyck et al. (2011b) [217] | 20-65 |  | ✓ | Neighborhood Environment Walkability Scale (NEWS) | Subjective | ✓ |  |
|  |  | Van Dyck et al. (2011c) [218] | 20-65 |  | ✓ | Geographic Information Systems (GIS) | Objective | ✓ |  |
|  |  | Van Dyck et al. (2011d) [219] | 20-65 |  | ✓ | Geographic Information Systems (GIS) | Objective | ✓ |  |
|  |  | Van Tuyckom (2011) [220] | 15+ | ✓ |  |  |  |  |  |
|  |  | Vandenbulcke et al. (2009) [221] | 18-65 | ✓ |  |  |  |  |  |
|  |  | Vandenbulcke (2011) [222] | 25+ but includes category <25 which is just defined as those who are working & <25 | ✓ |  |  |  |  |  |
| Croatia | 1 | Milošević et al. (2009) [223] | 18+ | ✓ |  |  |  |  |  |
| Czech Republic | 3 | Dygryn et al. (2010) [224] | 20-64 |  | ✓ | Geographic Information Systems (GIS) | Objective | ✓ |  |
|  |  | Frömel et al. (2009) [225] | 15+ | ✓ |  |  |  | ✓ |  |
|  |  | Sigmundová et al. (2011) [226] | 15+ |  | ✓ | Abbreviated Neighborhood Environment Walkability Scale (ANEWS) | Subjective | ✓ |  |
| Denmark | 1 | Toftager et al. (2011) [227] | 16+ | ✓ |  |  |  |  |  |
| France | 1 | Bertrais et al. (2004) [228] | 45-68 | ✓ |  |  |  |  |  |
| Greece | 1 | Pitsavos et al. (2005) [229] | 20-89 | ✓ |  |  |  |  |  |
| Italy | 1 | Bonnefoy et al. (2003) [230] | 20+ | ✓ |  |  |  |  |  |
| Netherlands | 9 | Engbers and Hendriksen (2010) [231] | 18-65 | ✓ |  |  |  |  |  |
|  |  | Gast et al. (2007) please check year [232] | 20-69 | ✓ |  |  |  |  |  |
|  |  | Kamphuis et al. (2008) [233] | 25-75 | ✓ |  |  |  |  |  |
|  |  | Keijer & Rietveld (2000) [234] | 0+ (Household data) | ✓ |  |  |  |  |  |
|  |  | Maas et al. (2008) [235] | 12+ | ✓ |  |  |  |  |  |
|  |  | Maat & Timmermans (2009) [236] | 18+ |  | ✓ | Geographic Information Systems (GIS) | Objective | ✓ |  |
|  |  | van Lenthe et al. (2005) [237] | 20-69 |  | ✓ | Geographic Information Systems (GIS) | Objective | ✓ |  |
|  |  | Wendel-Vos et al. (2004) [238] | 20-59 |  | ✓ | Geographic Information Systems (GIS) | Objective | ✓ |  |
|  |  | Wendel-Vos et al. (2008) [239] | 18+ | ✓ |  |  |  |  |  |
| Poland | 1 | Kwaśniewska et al. (2010) [240] | 20-74 | ✓ |  |  |  |  |  |
| Portugal | 3 | Santana et al. (2009) [241] | 18+ |  | ✓ | Self-created index | Objective | ✓ |  |
|  |  | Santos et al. (2009) [242] | 18+ |  | ✓ | International Physical Activity Questionnaire Environmental Module (IPAQ-E) | Subjective | ✓ |  |
|  |  | Santos et al. (2008) [243] | 18+ |  | ✓ | International Physical Activity Questionnaire Environmental Module (IPAQ-E) | Subjective | ✓ |  |
| Spain | 3 | Bolívar et al. (2010) [244] | 16+ | ✓ |  |  |  |  |  |
|  |  | Molina-García et al. (2010) [245] | Students (Mean: 22.4; SD: 5.3) |  | ✓ | Neighborhood Environment Walkability Scale (NEWS) | Subjective | ✓ |  |
|  |  | Pascual et al. (2009) [246] | 25-74 | ✓ |  |  |  |  |  |
| Sweden | 5 | Bergman et al. (2008) [247] | 18-74 | ✓ |  |  |  |  |  |
|  |  | Bergman et al. (2009) [248] | 18-74 |  | ✓ | International Physical Activity Questionnaire Environmental Module (IPAQ-E) | Subjective | ✓ |  |
|  |  | Björk et al. (2008) [249] | 18-80 |  | ✓ | Geographic Information Systems (GIS) | Objective | ✓ |  |
|  |  | Gidlöf-Gunnarsson & Ohrstrom (2007) [250] | 18-75 | ✓ |  |  |  |  |  |
|  |  | Sundquist et al. (2011) [251] | 20-65 |  | ✓ | Geographic Information Systems (GIS) | Objective | ✓ |  |
| United Kingdom | 16 | Cochrane et al. (2009) [252] | 16+ |  | ✓ | Geographic Information Systems (GIS) & Abbreviated Neighborhood Environment Walkability Scale (ANEWS) | Both | ✓ |  |
|  |  | Coombes et al. (2010) [133] | 16+ |  | ✓ | Geographic Information Systems (GIS) | Objective | ✓ |  |
|  |  | Foster et al. (2004) [253] | 16-74 |  | ✓ | Self-created items | Subjective | ✓ |  |
|  |  | Foster et al. (2009) [254] | 45-74 |  | ✓ | Geographic Information Systems (GIS) | Objective | ✓ |  |
|  |  | Foster et al. (2011) [255] | 41+ | ✓ |  |  |  |  |  |
|  |  | Harrison et al. (2007) [256] | Mean: 49.8 (SD: 17.6) | ✓ |  |  |  |  |  |
|  |  | Hillsdon et al. (2006) [257] | 40-70 |  | ✓ | Geographic Information Systems (GIS) | Objective | ✓ |  |
|  |  | Jones et al. (2009) [258] | 16+ |  | ✓ | Geographic Information Systems (GIS) | Objective | ✓ |  |
|  |  | Mason et al. (2011) [259] | 18+ |  | ✓ | Self-created items | Both | ✓ |  |
|  |  | Ogilvie et al. (2008) [260] | 16+ |  | ✓ | Geographic Information Systems (GIS) | Objective | ✓ |  |
|  |  | Panter et al. (2008a) [261] | 16+ |  | ✓ | Geographic Information Systems (GIS) & Abbreviated Neighborhood Environment Walkability Scale (ANEWS) | Both | ✓ |  |
|  |  | Panter & Jones (2008b) [262] | 16+ |  | ✓ | Geographic Information Systems (GIS) & Abbreviated Neighborhood Environment Walkability Scale (ANEWS) | Both | ✓ |  |
|  |  | Panter et al. (2011) [263] | 45-74 |  | ✓ | Geographic Information Systems (GIS) | Objective | ✓ |  |
|  |  | Parkes & Kearns (2006) [264] | 16+ | ✓ |  |  |  |  |  |
|  |  | Parkin et al. (2008) [265] | 16+ | ✓ |  |  |  |  |  |
|  |  | Stafford et al. (2007) [266] | 16+ | ✓ |  |  |  |  |  |
| Multi-country studies | 8 | Bamana et al. (2008) [267] | 18+ |  | ✓ | Self-created items | Subjective | ✓ |  |
|  |  | Ellaway et al. (2005) [268] | 20+ |  | ✓ | Self-created items | Both | ✓ |  |
|  |  | Guthold et al. (2008) [269] | 18-69 | ✓ |  |  |  |  |  |
|  |  | Miles (2008) [270] | 20+ | ✓ |  |  |  |  |  |
|  |  | Rütten et al. (2001) [271] | 18+ | ✓ |  |  |  |  |  |
|  |  | Rütten & Abu-Omar (2004) [272] | 15+ | ✓ |  |  |  |  |  |
|  |  | Shenassa et al. (2006) [273] | 20+ | ✓ |  |  |  |  |  |
|  |  | Ståhl et al. (2001) [274] | 18+ | ✓ |  |  |  |  |  |
| **Zapata-Diamedi & Veerman (2016)** [275] **23** | | | | | | | | | |
| Australia | 23 | Astell-Burt et al. (2014) [276] | 45+ | ✓ |  |  |  |  |  |
|  |  | Astell-Burt et al. (2015) [277] | 45+ | ✓ |  |  |  |  |  |
|  |  | Christian et al. (2011) [278] | 18+ |  | ✓ | Walkability Index (Geographic Information Systems) | Objective | ✓ |  |
|  |  | Cleland et al. (2013) [279] | 18-45 |  | ✓ | Self-created items | Subjective | ✓ |  |
|  |  | Duncan et al. (2010) [280] | 20-65 |  | ✓ | Neighborhood Environment Walkability Scale (NEWS) | Subjective | ✓ |  |
|  |  | Foster et al. (2014) [281] | 25-65 |  | ✓ | Geographic Information Systems (GIS) | Objective | ✓ |  |
|  |  | Giles-Corti et al. (2013) [282] | 18+ |  | ✓ | Geographic Information Systems (GIS) & Neighborhood Environment Walkability Scale (NEWS) | Both | ✓ |  |
|  |  | Heesch et al. (2014) [283] | 40-65 |  | ✓ | Neighborhood Environment Walkability Scale (ANEWS) | Subjective | ✓ |  |
|  |  | Knuiman et al. (2014) [284] | 18+ |  | ✓ | Geographic Information Systems (GIS) & Neighborhood Environment Walkability Scale (NEWS) | Both | ✓ |  |
|  |  | Koohsari et al. (2013a) [285] | 18+ |  | ✓ | Geographic Information Systems (GIS) & Neighborhood Environment Walkability Scale (NEWS) | Both | ✓ |  |
|  |  | Koohsari et al. (2013b) [286] | 18+ |  | ✓ | Geographic Information Systems (GIS) & Neighborhood Environment Walkability Scale (NEWS) | Both | ✓ |  |
|  |  | Koohsari et al. (2014) [287] | 20-65 |  | ✓ | Geographic Information Systems (GIS) | Objective | ✓ |  |
|  |  | Learnihan et al. (2011) [288] | 18+ |  | ✓ | Geographic Information Systems (GIS) & Neighborhood Environment Walkability Scale (NEWS) | Both | ✓ |  |
|  |  | McCormack et al. (2012) [289] | 18+ |  | ✓ | Geographic Information Systems (GIS) | Objective | ✓ |  |
|  |  | McKibbin (2011) [290] | NA^5^ |  | ✓ | Geographic Information Systems (GIS) | Objective | ✓ |  |
|  |  | Owen et al. (2010) [129] | 18-65 |  | ✓ | Geographic Information Systems (GIS) & Neighborhood Environment Walkability Scale (NEWS) | Both | ✓ |  |
|  |  | Shimura et al. (2012) [291] | 50-65 |  | ✓ | Geographic Information Systems (GIS) | Objective | ✓ |  |
|  |  | Sugiyama et al. (2014) [292] | 18-66 |  | ✓ | Neighborhood Environment Walkability Scale (ANEWS) | Subjective | ✓ |  |
|  |  | Sugiyama et al. (2013) [293] | 20-65 |  | ✓ | Geographic Information Systems (GIS) | Objective | ✓ |  |
|  |  | Sugiyama et al. (2009) [294] | 20-65 |  | ✓ | Neighborhood Environment Walkability Scale (ANEWS) | Subjective | ✓ |  |
|  |  | Titze et al. (2010) [295] | 18-78 |  | ✓ | Neighborhood Environment Walkability Scale (ANEWS) | Subjective | ✓ |  |
|  |  | Villanueva et al. (2014) [14] | 18+ |  | ✓ | Geographic Information Systems (GIS) | Objective | ✓ |  |
|  |  | Wilson et al. (2011) [296] | 40-65 |  | ✓ | Geographic Information Systems (GIS) | Objective | ✓ |  |

**1. Duplicates included**

**2. NA - Unable to determine from the information provided in the article if walkability was assessed**

**3. NA - Was unable to retrieve the article**

**4. NA - Was unable to retrieve measures or items of walkability**

**5. NA - Age not collected or reported in the article**

**6. Subjective measures of walkability were defined as ones in which participants were asked to rate or provide their own perceptions of whether or not the neighbourhoods they lived in were walkable or not. Objective measures are ones in which the researchers calculated the walkability of participants’ neighbourhoods or used information from external sources (for example Geographic Information Systems) to determine neighbourhood walkability.**

**References**

1. Barnett DW, Barnett A, Nathan A, Van Cauwenberg J, Cerin E. Built environmental correlates of older adults’ total physical activity and walking: a systematic review and meta-analysis. Int J Behav Nutr Phys Act. 2017;14:103.

2. Aird RL, Buys L. Active aging: exploration into self-ratings of “being active,” out-of-home physical activity, and participation among older australian adults living in four different settings. Journal of Aging Research. 2015; doi:10.1155/2015/501823.

3. Bird S, Radermacher H, Feldman S, Sims J, Kurowski W, Browning C, Thomas S. Factors influencing the physical activity levels of older people from culturally-diverse communities: an Australian experience. Ageing Soc Camb. 2009;29:1275–94.

4. Bird SR, Radermacher H, Sims J, Feldman S, Browning C, et al. Factors affecting walking activity of older people from culturally diverse groups: an Australian experience. J Sci Med Sport. 2010;13:417–23.

5. Cerin E, Rainey-Smith SR, Ames D, Lautenschlager NT, Macaulay SL, Fowler C, et al. Associations of neighborhood environment with brain imaging outcomes in the Australian Imaging, Biomarkers and Lifestyle cohort. Alzheimer’s & Dementia. 2017;13:388–98.

6. Espinel PT, Chau JY, van der Ploeg HP, Merom D. Older adults’ time in sedentary, light and moderate intensity activities and correlates: Application of Australian time use survey. J Sci Med Sport. 2015;18:161–6.

7. Lim K, Taylor L. Factors associated with physical activity among older people—a population-based study. Prev Med. 2005;40:33–40.

8. Macniven R, Pye V, Merom D, Milat A, Monger C, Bauman A, et al. Barriers and enablers to physical activity among older Australians who want to increase their physical activity levels. J Phys Act Health. 2014;11:1420–9.

9. Merom D, Gebel K, Fahey P, Astell-Burt T, Voukelatos A, Rissel C, et al. Neighborhood walkability, fear and risk of falling and response to walking promotion: The easy steps to health 12-month randomized controlled trial. Prev Med Rep. 2015;2 Suppl C:704–10.

10. Nathan A, Pereira G, Foster S, Hooper P, Saarloos D, Giles-Corti B. Access to commercial destinations within the neighbourhood and walking among Australian older adults. Int J Behav Nutr Phys Act. 2012;9:133.

11. Nathan A, Wood L, Giles-Corti B. Perceptions of the built environment and associations with walking among retirement village residents. Environ Behav. 2014;46:46–69.

12. Nathan A, Wood L, Giles-Corti B. Exploring socioecological correlates of active living in retirement village residents. J Aging Phys Act. 2014;22:1–15.

13. Nathan A, Wood L, Giles-Corti B. Examining correlates of self-reported and objectively measured physical activity among retirement village residents. Australas J Ageing. 2014;33:250–6.

14. Villanueva K, Knuiman M, Nathan A, Giles-Corti B, Christian H, Foster S, et al. The impact of neighborhood walkability on walking: Does it differ across adult life stage and does neighborhood buffer size matter? Health Place. 2014;25 Suppl C:43–6.

15. Van Cauwenberg J, Van Holle V, De Bourdeaudhuij I, Van Dyck D, Deforche B. Neighborhood walkability and health outcomes among older adults: The mediating role of physical activity. Health Place. 2016;37 Suppl C:16–25.

16. Holle VV, Cauwenberg JV, Gheysen F, Dyck DV, Deforche B, Weghe NV de, et al. The association between Belgian older adults’ physical functioning and physical activity: What is the moderating role of the physical environment? PLOS ONE. 2016;11:e0148398.

17. Salvador EP, Reis RS, Florindo AA. Practice of walking and its association with perceived environment among elderly Brazilians living in a region of low socioeconomic level. Int J Behav Nutr Phys Act. 2010;7:67.

18. Chad KE, Reeder BA, Harrison EL, Ashworth NL, Sheppard SM, Schultz SL, et al. Profile of physical activity levels in community-dwelling older adults. Med Sci Sports Exerc. 2005;37:1774–84.

19. Chaudhury H, Campo M, Michael Y, Mahmood A. Neighbourhood environment and physical activity in older adults. Soc Sci Med. 2016;149 Suppl C:104–13.

20. de Melo LL: PhD thesis. Perceived neighbourhood environment and health-related outcomes among older adults. 2013, University of Manitoba: Faculty of applied health sciences, https://mspace.lib.umanitoba.ca/bitstream/handle/1993/22258/de%20Melo_Lucelia.pdf.pdf?sequence=1. Accessed 5 Feb 2018.

21. de Melo LL, Menec V, Porter MM, Ready AE. Personal factors, perceived environment, and objectively measured walking in old age. J Aging Phys Act. 2010;18:280–92.

22. Gauvin L, Richard L, Kestens Y, Shatenstein B, Daniel M, Moore SD, et al. Living in a well-serviced urban area is associated with maintenance of frequent walking among seniors in the VoisiNuAge Study. J Gerontol Ser B. 2012;67B:76–88.

23. Hirsch JA, Winters M, Ashe MC, Clarke PJ, McKay HA. Destinations that older adults experience within their GPS activity spaces: Relation to objectively measured physical activity. Environ Behav. 2016;48:55–77.

24. Julien D, Richard L, Gauvin L, Fournier M, Kestens Y, Shatenstein B, et al. Transit use and walking as potential mediators of the association between accessibility to services and amenities and social participation among urban-dwelling older adults: Insights from the VoisiNuAge study. J Transp Health. 2015;2:35–43.

25. Pan SY, Cameron C, Des Meules M, Morrison H, Craig CL, Jiang X. Individual, social, environmental, and physical environmental correlates with physical activity among Canadians: A cross-sectional study. BMC Public Health. 2009;9:21.

26. Spinney JEL, Millward H. Active living among older Canadians: A time-use perspective over 3 decades. J Aging Phys Act. 2014;22:103–13.

27. Winters M, Barnes R, Venners S, Ste-Marie N, McKay H, Sims-Gould J, et al. Older adults’ outdoor walking and the built environment: does income matter? BMC Public Health. 2015;15:876.

28. Zhang Y, Li Y, Liu Q, Li C. The built environment and walking activity of the elderly: an empirical analysis in the Zhongshan metropolitan area, China. Sustainability. 2014;6:1076–92.

29. Gómez LF, Parra DC, Buchner D, Brownson RC, Sarmiento OL, Pinzón JD, et al. Built environment attributes and walking patterns among the elderly population in Bogotá. Am J Prev Med. 2010;38:592–9.

30. Pelclová J, Frömel K, Bláha L, Zając-Gawlak I, Tlučáková L. Neighborhood environment and walking for transport and recreation in Central European older adults. Acta Gymnica. 2012;42:49–56.

31. Cerin E, Zhang CJP, Barnett A, Sit CHP, Cheung MMC, Johnston JM, et al. Associations of objectively-assessed neighborhood characteristics with older adults’ total physical activity and sedentary time in an ultra-dense urban environment: Findings from the ALECS study. Health Place. 2016;42 Suppl C:1–10.

32. Cerin E, Mellecker R, Macfarlane DJ, Barnett A, Cheung M, Sit CHP, et al. Socioeconomic status, neighborhood characteristics, and walking within the neighborhood among older Hong Kong Chinese. J Aging Health. 2013;25:1425–44.

33. Arnadottir SA, Gunnarsdottir ED, Lundin-Olsson L. Are rural older Icelanders less physically active than those living in urban areas? A population-based study. Scand J Public Health. 2009;37:409–17.

34. Lotfi S, Koohsari MJ. Neighborhood walkability in a city within a developing country. J Urban Plan Dev. 2011;137:402–8.

35. McKee G, Kearney PM, Kenny RA. The factors associated with self-reported physical activity in older adults living in the community. Age Ageing. 2015;44:586–92.

36. Murtagh EM, Murphy MH, Murphy NM, Woods C, Nevill AM, Lane A. Prevalence and correlates of physical inactivity in community-dwelling older adults in Ireland. PLOS ONE. 2015;10:e0118293.

37. Carvalho Sampaio RA, Sewo Sampaio PY, Yamada M, Ogita M, Arai H. Urban-rural differences in physical performance and health status among older Japanese community-dwelling women. J Clin Gerontol Geriatr. 2012;3:127–31.

38. Chen T, Lee JS, Kawakubo K, Watanabe E, Mori K, Kitaike T, et al. Features of perceived neighborhood environment associated with daily walking time or habitual exercise: Differences across gender, age, and employment status in a community–dwelling population of Japan. Environ Health Prev Med. 2013;18:368–76.

39. Hanibuchi T, Kawachi I, Nakaya T, Hirai H, Kondo K. Neighborhood built environment and physical activity of Japanese older adults: Results from the Aichi Gerontological Evaluation Study (AGES). BMC Public Health. 2011;11:657.

40. Inoue S, Ohya Y, Odagiri Y, Takamiya T, Kamada M, Okada S, et al. Perceived neighborhood environment and walking for specific purposes among elderly Japanese. J Epidemiol Fukuoka. 2011;21:481–90.

41. Sewo Sampaio PY, Ito E, Carvalho Sampaio RA. The association of activity and participation with quality of life between Japanese older adults living in rural and urban areas. J Clin Gerontol Geriatr. 2013;4:51–6.

42. Tanaka T, Tanaka K, Suyama K, Honda S, Senjyu H, Kozu R. A comparison of objective physical activity, muscle strength, and depression among community-dwelling older women living in sloped versus non-sloped environments. J Nutr Health Aging. 2016;20:520–4.

43. Tsunoda K, Tsuji T, Kitano N, Mitsuishi Y, Yoon J-Y, Yoon J, et al. Associations of physical activity with neighborhood environments and transportation modes in older Japanese adults. Prev Med. 2012;55:113–8.

44. Baceviciene M, Alisauskas J. Perceived constraints on exercise in the group of the elderly: a pilot study. Cent Eur J Med. 2013;8:689–95.

45. Kaur J, Kaur G, Ho BK, Yao WK, Salleh M, Lim KH. Predictors of physical inactivity among elderly Malaysians: Recommendations for policy planning. Asia Pac J Public Health. 2015;27:314–22.

46. Böcker L, Amen P van, Helbich M. Elderly travel frequencies and transport mode choices in Greater Rotterdam, the Netherlands. Transportation. 2017;44:831–52.

47. Jongeneel-Grimen B, Busschers W, Droomers M, Oers HAM van, Stronks K, Kunst AE. Change in neighborhood traffic safety: Does it matter in terms of physical activity? PLOS ONE. 2013;8:e62525.

48. Jongeneel-Grimen B, Droomers M, van Oers HAM, Stronks K, Kunst AE. The relationship between physical activity and the living environment: A multi-level analyses focusing on changes over time in environmental factors. Health Place. 2014;26 Suppl C:149–60.

49. Timmermans EJ, Schaap LA, Visser M, van der Ploeg HP, Wagtendonk AJ, van der Pas S, et al. The association of the neighbourhood built environment with objectively measured physical activity in older adults with and without lower limb osteoarthritis. BMC Public Health. 2016;16:710.

50. Piro FN, Nœss Ø, Claussen B. Physical activity among elderly people in a city population: The influence of neighbourhood level violence and self perceived safety. J Epidemiol Community Health. 2006;60:626–32.

51. Koh PP, Leow BW, Wong YD. Mobility of the elderly in densely populated neighbourhoods in Singapore. Sustain Cities Soc. 2015;14 Suppl C:126–32.

52. Kolbe-Alexander TL, Pacheco K, Tomaz SA, Karpul D, Lambert EV. The relationship between the built environment and habitual levels of physical activity in South African older adults: a pilot study. BMC Public Health. 2015;15:518.

53. Lee H-S, Park E-Y. Associations of neighborhood environment and walking in Korean elderly women: a comparison between urban and rural dwellers. Asian Women. 2015;31:1–21.

54. Yeom H-A, Jung D, Choi M. Adherence to physical activity among older adults using a geographic information system: Korean National Health and Nutrition Examinations Survey IV. Asian Nurs Res. 2011;5:118–27.

55. Asawachaisuwikrom W: PhD thesis. Predictors of physical activity among older Thai adults. 2001. University of Texas at Austin: Faculty of the graduate school. https://repositories.lib.utexas.edu/handle/2152/1683. Accessed 8 Feb 2018.

56. Davis MG, Fox KR, Hillsdon M, Coulson JC, Sharp DJ, Stathi A, et al. Getting out and about in older adults: the nature of daily trips and their association with objectively assessed physical activity. Int J Behav Nutr Phys Act. 2011;8:116.

57. Fox KR, Hillsdon M, Sharp D, Cooper AR, Coulson JC, Davis M, et al. Neighbourhood deprivation and physical activity in UK older adults. Health Place. 2011;17:633–40.

58. Jefferis BJ, Sartini C, Lee I-M, Choi M, Amuzu A, Gutierrez C, et al. Adherence to physical activity guidelines in older adults, using objectively measured physical activity in a population-based study. BMC Public Health. 2014;14:382.

59. McMurdo MET, Argo I, Crombie IK, Feng Z, Sniehotta FF, Vadiveloo T, et al. Social, environmental and psychological factors associated with objective physical activity levels in the over 65s. Plos One. 2012;7:e31878.

60. Persson A, While A. Physical activity among older people and related factors. Health Educ J. 2012;71:144–53.

61. Sugiyama T, Ward Thompson C. Older people’s health, outdoor activity and supportiveness of neighbourhood environments. Landsc Urban Plan. 2007;83:168–75.

62. Sugiyama T, Leslie E, Giles-Corti B, Owen N. Physical activity for recreation or exercise on neighbourhood streets: Associations with perceived environmental attributes. Health Place. 2009;15:1058–63.

63. Sniehotta FF, Gellert P, Witham MD, Donnan PT, Crombie IK, McMurdo ME. Psychological theory in an interdisciplinary context: psychological, demographic, health-related, social, and environmental correlates of physical activity in a representative cohort of community-dwelling older adults. Int J Behav Nutr Phys Act. 2013;10:106.

64. Thompson CW, Curl A, Aspinall P, Alves S, Zuin A. Do changes to the local street environment alter behaviour and quality of life of older adults? The ‘DIY Streets’ intervention. Br J Sports Med. 2012; http://dx.doi.org/10.1136/bjsports-2012-091718.

65. Thompson JL, Bentley G, Davis M, Coulson J, Stathi A, Fox KR. Food shopping habits, physical activity and health-related indicators among adults aged ≥70 years. Public Health Nutr. 2011;14:1640–9.

66. Bracy NL, Millstein RA, Carlson JA, Conway TL, Sallis JF, Saelens BE, et al. Is the relationship between the built environment and physical activity moderated by perceptions of crime and safety? Int J Behav Nutr Phys Act. 2014;11:24.

67. Cain KL, Millstein RA, Sallis JF, Conway TL, Gavand KA, Frank LD, et al. Contribution of streetscape audits to explanation of physical activity in four age groups based on the Microscale Audit of Pedestrian Streetscapes (MAPS). Soc Sci Med. 2014;116 Suppl C:82–92.

68. Carlson JA, Bracy NL, Sallis JF, Millstein RA, Saelens BE, Kerr J, et al. Sociodemographic moderators of relations of neighborhood safety to physical activity. Med Sci Sports Exerc. 2014;46:1554–63.

69. Carlson JA, Sallis JF, Conway TL, Saelens BE, Frank LD, Kerr J, et al. Interactions between psychosocial and built environment factors in explaining older adults’ physical activity. Prev Med. 2012;54:68–73.

70. Ding D, Sallis JF, Norman GJ, Frank LD, Saelens B, Kerr J, et al. Neighborhood environment and physical activity among older adults: Do the relationships differ by driving status? J Aging Phys Act. 2014;22:421–31.

71. Frank L, Kerr J, Rosenberg D, King A. Healthy aging and where you live: Community design relationships with physical activity and body weight in older Americans. J Phys Act Health. 2010;7:S82–90.

72. Gallagher NA, Clarke PJ, Ronis DL, Cherry CL, Nyquist L, Gretebeck KA. Influences on neighborhood walking in older adults. Res Gerontol Nurs. 2012;5:238–50.

73. Grant-Savela SD. Active living among older residents of a rural naturally occurring retirement community. J Appl Gerontol. 2010;29:531–53.

74. Hall KS, McAuley E. Individual, social environmental and physical environmental barriers to achieving 10000 steps per day among older women. Health Educ Res. 2010;25:478–88.

75. Han B, Sadarangani T, Wyatt LC, Zanowiak JM, Kwon SC, Trinh-Shevrin C, et al. Correlates of physical activity among middle-aged and older Korean Americans at risk for diabetes. J Nurs Scholarsh. 2016;48:48–57.

76. Hannon L, Sawyer P, Allman RM. Housing, the neighborhood environment, and physical activity among older African Americans. J Health Disparities Res Pract. 2012;5:27–41.

77. James P, Troped PJ, Hart JE, Joshu CE, Colditz GA, Brownson RC, et al. Urban sprawl, physical activity, and body mass index: nurses’ health study and nurses’ health study II. Am J Public Health. 2013;103:369–75.

78. Kerr J, Carlson JA, Sallis JF, Rosenberg D, Leak CR, Saelens BE, et al. Assessing health-related resources in senior living residences. J Aging Stud. 2011;25:206–14.

79. King AC, Toobert D, Ahn D, Resnicow K, Coday M, Riebe D, et al. Perceived environments as physical activity correlates and moderators of intervention in five studies. Am J Health Promot. 2006;21:24–35.

80. King WC, Brach JS, Belle S, Killingsworth R, Fenton M, Kriska AM. The relationship between convenience of destinations and walking levels in older women. Am J Health Promot. 2003;18:74–82.

81. Latham K, Williams MM. Does neighborhood disorder predict recovery from mobility limitation? Findings from the Health and Retirement Study. J Aging Health. 2015;27:1415–42.

82. Lee I-M, Ewing R, Sesso HD. The built environment and physical activity levels: the Harvard alumni health study. Am J Prev Med. 2009;37:293–8.

83. Li Y, Kao D, Dinh TQ. Correlates of neighborhood environment with walking among older Asian Americans. J Aging Health. 2015;27:17–34.

84. Li F, Fisher KJ, Brownson RC, Bosworth M. Multilevel modelling of built environment characteristics related to neighbourhood walking activity in older adults. J Epidemiol Community Health. 2005;59:558–64.

85. Li Y, Kao D, Dinh TQ. Correlates of neighborhood environment with walking among older Asian Americans. J Aging Health. 2015;27:17–34.

86. Maisel JL. Impact of older adults’ neighborhood perceptions on walking behavior. J Aging Phys Act. 2016;24:247–55.

87. Leon CFM de, Cagney KA, Bienias JL, Barnes LL, Skarupski KA, Scherr PA, et al. Neighborhood social cohesion and disorder in relation to walking in community-dwelling older adults: a multilevel analysis. J Aging Health. 2009;21:155–71.

88. Morris MB, Chapula BT, Chi BH, Mwango A, Chi HF, Mwanza J, et al. Use of task-shifting to rapidly scale-up HIV treatment services: Experiences from Lusaka, Zambia. BMC Health Serv Res. 2009;9:5.

89. Michael Y, Beard T, Choi D, Farquhar S, Carlson N. Measuring the influence of built neighborhood environments on walking in older adults. J Aging Phys Act. 2006;14:302–12.

90. Michael YL, Perdue LA, Orwoll ES, Stefanick ML, Marshall LM. Physical activity resources and changes in walking in a cohort of older men. Am J Public Health. 2010;100:654–60.

91. Mowen A, Orsega-Smith E, Payne L, Ainsworth B, Godbey G. The role of park proximity and social support in shaping park visitation, physical activity, and perceived health among older adults. J Phys Act Health. 2007;4:167–79.

92. Nagel CL, Carlson NE, Bosworth M, Michael YL. The relation between neighborhood built environment and walking activity among older adults. Am J Epidemiol. 2008;168:461–8.

93. Perry CK, Herting JR, Berke EM, Nguyen HQ, Vernez Moudon A, Beresford SAA, et al. Does neighborhood walkability moderate the effects of intrapersonal characteristics on amount of walking in post-menopausal women? Health Place. 2013;21 Suppl C:39–45.

94. Procter-Gray E, Leveille SG, Hannan MT, Cheng J, Kane K, Li W. Variations in community prevalence and determinants of recreational Vs. utilitarian walking in older age. Gerontologist. 2015;55:321–2.

95. Satariano WA, Ivey SL, Kurtovich E, Kealey M, Hubbard AE, Bayles CM, et al. Lower-body function, neighborhoods, and walking in an older population. Am J Prev Med. 2010;38:419–28.

96. Shores KA, West ST, Theriault DS, Davison EA. Extra-individual correlates of physical activity attainment in rural older adults. J Rural Health. 2009;25:211–8.

97. Shin W-H, Kweon B-S, Shin W-J. The distance effects of environmental variables on older African American women’s physical activity in Texas. Landsc Urban Plan. 2011;103:217–29.

98. Towne SD, Won J, Lee S, Ory MG, Forjuoh SN, Wang S, et al. Using walk score^tm^ and neighborhood perceptions to assess walking among middle-aged and older adults. J Community Health N Y. 2016;41:977–88.

99. Troped PJ, Starnes HA, Puett RC, Tamura K, Cromley EK, James P, et al. Relationships between the built environment and walking and weight status among older women in three U.S. States. J Aging Phys Act. 2014;22:114–25.

100. Wang Z, Lee C. Site and neighborhood environments for walking among older adults. Health Place. 2010;16:1268–79.

101. Wilcox S, Bopp M, Oberrecht L, Kammermann SK, McElmurray CT. Psychosocial and perceived environmental correlates of physical activity in rural and older African American and White Women. J Gerontol Ser B. 2003;58:P329–37.

102. Cerin E, Nathan A, van Cauwenberg J, Barnett DW, Barnett A. The neighbourhood physical environment and active travel in older adults: a systematic review and meta-analysis. Int J Behav Nutr Phys Act. 2017;14:15.

103. Boruff BJ, Nathan A, Nijënstein S. Using GPS technology to (re)-examine operational definitions of ‘neighbourhood’ in place-based health research. Int J Health Geogr. 2012;11:22.

104. Garrard J: Senior Victorians and walking: Obstacles and opportunities. 2013. https://trid.trb.org/view/1286948. Accessed 5 Feb 2018.

105. Van Cauwenberg J, Clarys P, De Bourdeaudhuij I, Van Holle V, Verté D, De Witte N, et al. Physical environmental factors related to walking and cycling in older adults: the Belgian aging studies. BMC Public Health. 2012;12:142.

106. Van Holle V, Van Cauwenberg J, Van Dyck D, Deforche B, Van de Weghe N, De Bourdeaudhuij I. Relationship between neighborhood walkability and older adults’ physical activity: results from the Belgian Environmental Physical Activity Study in Seniors (BEPAS Seniors). Int J Behav Nutr Phys Act. 2014;11:110.

107. Corseuil MW, Schneider IJC, Silva DAS, Costa FF, Silva KS, Borges LJ, et al. Perception of environmental obstacles to commuting physical activity in Brazilian elderly. Prev Med. 2011;53:289–92.

108. Corseuil Giehl MW, Hallal PC, Corseuil CW, Schneider IJC, d’Orsi E. Built environment and walking behavior among Brazilian older adults: a population-based study. J Phys Act Health. 2016;13:617–24.

109. Corseuil Giehl MW, Hallal PC, Brownson RC, d’Orsi E. Exploring associations between perceived measures of the environment and walking among Brazilian older adults. J Aging Health. 2017;29:45–67.

110. Barnes R, Winters M, Ste-Marie N, McKay H, Ashe MC. Age and retirement status differences in associations between the built environment and active travel behaviour. J Transp Health. 2016;3:513–22.

111. Chudyk AM, Winters M, Moniruzzaman M, Ashe MC, Gould JS, McKay H. Destinations matter: The association between where older adults live and their travel behavior. J Transp Health. 2015;2:50–7.

112. Moniruzzaman M, Páez A, Nurul Habib KM, Morency C. Mode use and trip length of seniors in Montreal. J Transp Geogr. 2013;30 Suppl C:89–99.

113. Moniruzzaman M, Páez A, Scott D, Morency C. Trip generation of seniors and the geography of walking in Montreal. Environ Plan Econ Space. 2015;47:957–76.

114. Tsai L-T, Rantakokko M, Portegijs E, Viljanen A, Saajanaho M, Eronen J, et al. Environmental mobility barriers and walking for errands among older people who live alone vs. with others. BMC Public Health. 2013;13:1054.

115. Barnett A, Cerin E, Zhang CJP, Sit CHP, Johnston JM, Cheung MMC, et al. Associations between the neighbourhood environment characteristics and physical activity in older adults with specific types of chronic conditions: the ALECS cross-sectional study. Int J Behav Nutr Phys Act. 2016;13:53.

116. Cerin E, Macfarlane D, Sit CHP, Ho SY, Johnston JM, Chou KL, et al. Effects of built environment on walking among Hong Kong older adults. Hong Kong Med J. 2013;19 Suppl 4:39–41.

117. Cerin E, Lee K, Barnett A, Sit CH, Cheung M, Chan W, et al. Walking for transportation in Hong Kong Chinese urban elders: a cross-sectional study on what destinations matter and when. Int J Behav Nutr Phys Act. 2013;10:78.

118. Cerin E, Sit CH, Barnett A, Johnston JM, Cheung M-C, Chan W-M. Ageing in an ultra-dense metropolis: perceived neighbourhood characteristics and utilitarian walking in Hong Kong elders. Public Health Nutr Camb. 2014;17:225–32.

119. Etman A, Kamphuis CBM, Prins RG, Burdorf A, Pierik FH, van Lenthe FJ. Characteristics of residential areas and transportational walking among frail and non-frail Dutch elderly: does the size of the area matter? Int J Health Geogr. 2014;13:7.

120. Nyunt MSZ, Shuvo FK, Eng JY, Yap KB, Scherer S, Hee LM, et al. Objective and subjective measures of neighborhood environment (NE): relationships with transportation physical activity among older persons. Int J Behav Nutr Phys Act. 2015;12:108.

121. King D. Neighborhood and individual factors in activity in older adults: results from the neighborhood and senior health study. J Aging Phys Act. 2008;16:144–70.

122. King AC, Sallis JF, Frank LD, Saelens BE, Cain K, Conway TL, et al. Aging in neighborhoods differing in walkability and income: Associations with physical activity and obesity in older adults. Soc Sci Med. 2011;73:1525–33.

123. Mitchell AB: PhD thesis. The relationship of self-reported health status and perceived neighborhood built environment with the amount of self-reported walking among urban community dwelling older adults. Ph.D. University of Pennsylvania. 2012. https://search.proquest.com/docview/1171064153/abstract/9349032DA2054E1FPQ/1. Accessed 1 Feb 2018.

124. Patterson PK, Chapman NJ. Urban form and older residents’ service use, walking, driving, quality of life, and neighborhood satisfaction. Am J Health Promot. 2004;19:45–52.

125. Shigematsu R, Sallis JF, Conway TL, Saelens BE, Frank LD, Cain KL, et al. Age differences in the relation of perceived neighborhood environment to walking. Med Sci Sports Exerc. 2009;41:314–21.

126. Grasser G, Van Dyck D, Titze S, Stronegger W. Objectively measured walkability and active transport and weight-related outcomes in adults: a systematic review. J Public Health. 2013;58:615–25.

127. Cerin E, Leslie E, Toit L du, Owen N, Frank LD. Destinations that matter: Associations with walking for transport. Health Place. 2007;13:713–24.

128. Owen N, Cerin E, Leslie E, du Toit L, Coffee N, Frank LD, et al. Neighborhood walkability and the walking behavior of Australian adults. Am J Prev Med. 2007;33:387–95.

129. Owen N, De Bourdeaudhuij I, Sugiyama T, Leslie E, Cerin E, Van Dyck D, et al. Bicycle use for transport in an Australian and a Belgian city: Associations with built-environment attributes. J Urban Health. 2010;87:189–98.

130. Van Dyck D, Cardon G, Deforche B, Sallis JF, Owen N, De Bourdeaudhuij I. Neighborhood SES and walkability are related to physical activity behavior in Belgian adults. Prev Med. 2010;50 Suppl:S74–9.

131. Van Dyck D, Cerin E, Cardon G, Deforche B, Sallis JF, Owen N, et al. Physical activity as a mediator of the associations between neighborhood walkability and adiposity in Belgian adults. Health Place. 2010;16:952–60.

132. Pouliou T, Elliott SJ. Individual and socio-environmental determinants of overweight and obesity in urban Canada. Health Place. 2010;16:389–98.

133. Coombes E, Jones AP, Hillsdon M. The relationship of physical activity and overweight to objectively measured green space accessibility and use. Soc Sci Med. 2010;70:816–22.

134. Berke EM, Koepsell TD, Moudon AV, Hoskins RE, Larson EB. Association of the built environment with physical activity and obesity in older persons. Am J Public Health. 2007;97:486–92.

135. Bodea TD, Garrow LA, Meyer MD, Ross CL. Explaining obesity with urban form: a cautionary tale. Transp N Y. 2008;35:179–99.

136. Boer R, Zheng Y, Overton A, Ridgeway GK, Cohen DA. Neighborhood design and walking trips in ten U.S. metropolitan areas. Am J Prev Med. 2007;32:298–304.

137. Brown BB, Yamada I, Smith KR, Zick CD, Kowaleski-Jones L, Fan JX. Mixed land use and walkability: Variations in land use measures and relationships with BMI, overweight, and obesity. Health Place. 2009;15:1130–41.

138. Chatman DG. Residential choice, the built environment, and nonwork travel: evidence using new data and methods. Environ Plan A. 2009;41:1072–89.

139. Forsyth A, Oakes JM, Schmitz KH, Hearst M. Does residential density increase walking and other physical activity? Urban Stud. 2007;44:679–97.

140. Forsyth A, Hearst M, Oakes JM, Schmitz KH. Design and destinations: factors influencing walking and total physical activity. Urban Stud. 2008;45:1973–96.

141. Forsyth A, Michael Oakes J, Lee B, Schmitz KH. The built environment, walking, and physical activity: Is the environment more important to some people than others? Transp Res Part Transp Environ. 2009;14:42–9.

142. Frank LD, Andresen MA, Schmid TL. Obesity relationships with community design, physical activity, and time spent in cars. Am J Prev Med. 2004;27:87–96.

143. Frank LD, Sallis JF, Conway TL, Chapman JE, Saelens BE, Bachman W. Many pathways from land use to health: Associations between neighborhood walkability and active transportation, body mass index, and air quality. J Am Plann Assoc. 2006;72:75–87.

144. Frank LD, Saelens BE, Powell KE, Chapman JE. Stepping towards causation: Do built environments or neighborhood and travel preferences explain physical activity, driving, and obesity? Soc Sci Med. 2007;65:1898–914.

145. Frank LD, Kerr J, Sallis JF, Miles R, Chapman J. A hierarchy of sociodemographic and environmental correlates of walking and obesity. Prev Med. 2008;47:172–8.

146. Frank L, Kerr J, Saelens B, Sallis J, Glanz K, Chapman J. Food outlet visits, physical activity and body weight: variations by gender and race–ethnicity. British Journal of Sports Medicine. 2009;43:124–31.

147. Huang L, Stinchcomb DG, Pickle LW, Dill J, Berrigan D. Identifying clusters of active transportation using spatial scan statistics. Am J Prev Med. 2009;37:157–66.

148. Kitamura R, Mokhtarian P, Laidet L. A micro-analysis of land use and travel in five neighborhoods in the San Francisco Bay area. Transportation. 1997;24:125–58.

149. Lee C, Moudon AV. Correlates of walking for transportation or recreation purposes. J Phys Act Health. 2006;3:S77–98.

150. Li F, Harmer PA, Cardinal BJ, Bosworth M, Acock A, Johnson-Shelton D, et al. Built environment, adiposity, and physical activity in adults aged 50–75. Am J Prev Med. 2008;35:38–46.

151. Li F, Harmer P, Cardinal BJ, Bosworth M, Johnson-Shelton D, Moore JM, et al. Built environment and 1-year change in weight and waist circumference in middle-aged and older adults: Portland Neighborhood Environment and Health Study. Am J Epidemiol. 2009;169:401–8.

152. Lopez RP. Neighborhood risk factors for obesity. Obesity. 2007;15:2111–9.

153. McGinn AP, Evenson KR, Herring AH, Huston SL, Rodriguez DA. Exploring associations between physical activity and perceived and objective measures of the built environment. J Urban Health. 2007;84:162–84.

154. Oakes JM, Forsyth A, Schmitz KH. The effects of neighborhood density and street connectivity on walking behavior: the Twin Cities walking study. Epidemiol Perspect Innov. 2007;4:16.

155. Rundle A, Roux AVD, Freeman LM, Miller D, Neckerman KM, Weiss CC. The urban built environment and obesity in New York City: A multilevel analysis. Am J Health Promot. 2007;21 4 Suppl:326–34.

156. Rundle A, Neckerman KM, Freeman L, Lovasi GS, Purciel M, Quinn J, et al. Neighborhood food environment and walkability predict obesity in New York City. Environ Health Perspect. 2009;117:442–7.

157. Sallis JF, Saelens BE, Frank LD, Conway TL, Slymen DJ, Cain KL, et al. Neighborhood built environment and income: Examining multiple health outcomes. Soc Sci Med. 2009;68:1285–93.

158. Scott MM, Dubowitz T, Cohen DA. Regional differences in walking frequency and BMI: What role does the built environment play for Blacks and Whites? Health Place. 2009;15:897–902.

159. Smith KR, Brown BB, Yamada I, Kowaleski-Jones L, Zick CD, Fan JX. Walkability and body mass index: density, design, and new diversity measures. Am J Prev Med. 2008;35:237–44.

160. Zick CD, Smith KR, Fan JX, Brown BB, Yamada I, Kowaleski-Jones L. Running to the store? The relationship between neighborhood environments and the risk of obesity. Soc Sci Med. 2009;69:1493–500.

161. McCormack GR, Shiell A. In search of causality: a systematic review of the relationship between the built environment and physical activity among adults. Int J Behav Nutr Phys Act. 2011;8:125.

162. Macbeth AG. Bicycle lanes in Toronto. Inst Transp Eng ITE J Wash. 1999;69:38–46.

163. Meurs H, Haaijer R. Spatial structure and mobility. Transp Res Part Transp Environ. 2001;6:429–46.

164. Painter K. The influence of street lighting improvements on crime, fear and pedestrian street use, after dark. Landsc Urban Plan. 1996;35:193–201.

165. Bagley MN, Mokhtarian PL. The impact of residential neighborhood type on travel behavior: A structural equations modeling approach. Ann Reg Sci Heidelb. 2002;36:279–97.

166. Boone-Heinonen J, Guilkey DK, Evenson KR, Gordon-Larsen P. Residential self-selection bias in the estimation of built environment effects on physical activity between adolescence and young adulthood. Int J Behav Nutr Phys Act. 2010;7:70.

167. Boarnet MG, Greenwald M, McMillan TE. Walking, urban design, and health: toward a cost-benefit analysis framework. J Plan Educ Res. 2008;27:341–58.

168. Brown BB, Werner CM. Before and after a new light rail stop: resident attitudes, travel behavior, and obesity. J Am Plann Assoc. 2008;75:5–12.

169. Brown BB, Werner CM. A new rail stop: tracking moderate physical activity bouts and ridership. Am J Prev Med. 2007;33:306–9.

170. Burbidge S, Goulias K. Evaluating the impact of neighborhood trail development on active travel behavior and overall physical activity of suburban residents. Transp Res Rec J Transp Res Board. 2009;2135:78–86.

171. Cao X, Handy SL, Mokhtarian PL. The influences of the built environment and residential self-selection on pedestrian behavior: evidence from Austin, TX. Transp N Y. 2006;33:1–20.

172. Cao X, Mokhtarian PL, Handy SL. No particular place to go: an empirical analysis of travel for the sake of travel. Environ Behav. 2009;41:233–57.

173. Cao X (Jason), Mokhtarian PL, Handy SL. The relationship between the built environment and nonwork travel: A case study of Northern California. Transp Res Part Policy Pract. 2009;43:548–59.

174. Cao J. Exploring causal effects of neighborhood type on walking behavior using stratification on the propensity score. Environ and Planning A. 2010;42:287-504.

175. Cao X, Mokhtarian PL, Handy SL. Do changes in neighborhood characteristics lead to changes in travel behavior? A structural equations modeling approach. Transp N Y. 2007;34:535–56.

176. Cohen R, Lynch S, Bygrave H, Eggers E, Vlahakis N, Hilderbrand K, et al. Antiretroviral treatment outcomes from a nurse-driven, community-supported HIV/AIDS treatment programme in rural Lesotho: Observational cohort assessment at two years. J Int AIDS Soc. 2009;12:23.

177. Coogan PF, White LF, Adler TJ, Hathaway KM, Palmer JR, Rosenberg L. Prospective study of urban form and physical activity in the black women’s health study. Am J Epidemiol. 2009;170:1105–17.

178. Evenson KR, Herring AH, Huston SL. Evaluating change in physical activity with the building of a multi-use trail. Am J Prev Med. 2005;28:177–85.

179. Fitzhugh EC, Bassett DR, Evans MF. Urban trails and physical activity: a natural experiment. Am J Prev Med. 2010;39:259–62.

180. Greenwald M, Boarnet M. Built environment as determinant of walking behavior: analyzing nonwork pedestrian travel in Portland, Oregon. Transp Res Rec J Transp Res Board. 2001;1780:33–41.

181. Handy SL, Xinyu Cao, Mokhtarian PL. The causal influence of neighborhood design on physical activity within the neighborhood: evidence from Northern California. Am J Health Promot. 2008;22:350–8.

182. Handy S, Cao X, Mokhtarian PL. Self-selection in the relationship between the built environment and walking: empirical evidence from Northern California. J Am Plann Assoc. 2006;72:55–74.

183. Joh K, Boarnet M, Nguyen M, Fulton W, Siembab W, Weaver S. Accessibility, travel behavior, and new urbanism: case study of mixed-use centers and auto-oriented corridors in the South Bay region of Los Angeles, California. Transp Res Rec J Transp Res Board. 2008;2082:81–9.

184. Khattak AJ, Rodriguez D. Travel behavior in neo-traditional neighborhood developments: A case study in USA. Transp Res Part Policy Pract. 2005;39:481–500.

185. MacDonald JM, Stokes RJ, Cohen DA, Kofner A, Ridgeway GK. The effect of light rail transit on body mass index and physical activity. Am J Prev Med. 2010;39:105–12.

186. Pinjari AR, Bhat CR, Hensher DA. Residential self-selection effects in an activity time-use behavior model. Transp Res Part B Methodol. 2009;43:729–48.

187. Schwanen T, Mokhtarian PL. What if you live in the wrong neighborhood? The impact of residential neighborhood type dissonance on distance traveled. Transp Res Part Transp Environ. 2005;10:127–51.

188. Shay E, Fan Y, Rodríguez D, Khattak A. Drive or walk? Utilitarian trips within a neotraditional neighborhood. Transp Res Rec J Transp Res Board. 2006;1985:154–61.

189. Wells NM, Yang Y. Neighborhood design and walking: a quasi-experimental longitudinal study. Am J Prev Med. 2008;34:313–9.

190. Renalds AM, Smith THM, Hale PJ. A Systematic review of built environment and health. Family & Community Health. 2010;33:68–78.

191. Wood L, Shannon T, Bulsara M, Pikora T, McCormack G, Giles-Corti B. The anatomy of the safe and social suburb: An exploratory study of the built environment, social capital and residents’ perceptions of safety. Health Place. 2008;14:15–31.

192. Titze S, Stronegger WJ, Janschitz S, Oja P. Association of built-environment, social-environment and personal factors with bicycling as a mode of transportation among Austrian city dwellers. Prev Med. 2008;47:252–9.

193. Leyden KM. Social Capital and the Built Environment: The Importance of Walkable Neighborhoods. Am J Public Health. 2003;93:1546–51.

194. Dijkstra K, Pieterse ME, Pruyn A. Stress-reducing effects of indoor plants in the built healthcare environment: The mediating role of 194. Dijkstra K, Pieterse ME, Pruyn A. Stress-reducing effects of indoor plants in the built healthcare environment: The mediating role of perceived attractiveness. Prev Med. 2008;47:279–83.

195. Araya R, Dunstan F, Playle R, Thomas H, Palmer S, Lewis G. Perceptions of social capital and the built environment and mental health. Soc Sci Med. 2006;62:3072–83.

196. Abildso CG, Zizzi S, Abildso LC, Steele JC, Gordon PM. Built environment and psychosocial factors associated with trail proximity and use. Am J Health Behav Star City. 2007;31:374–83.

197. Bernstein KT, Galea S, Ahern J, Tracy M, Vlahov D. The built environment and alcohol consumption in urban neighborhoods. Drug Alcohol Depend. 2007;91:244–52.

198. Brown SC, Mason CA, Perrino T, Lombard JL, Martinez F, Plater-Zyberk E, et al. Built environment and physical functioning in Hispanic elders: the role of “eyes on the street.” Environ Health Perspect. 2008;116:1300–7.

199. Clarke P, Ailshire JA, Bader M, Morenoff JD, House JS. Mobility disability and the urban built environment. Am J Epidemiol. 2008;168:506–13.

200. Clarke P, George LK. The role of the built environment in the disablement process. Am J Public Health. 2005;95:1933–9.

201. Cohen DA, Inagami S, Finch B. The built environment and collective efficacy. Health Place. 2008;14:198–208.

202. Galea S, Ahern J, Rudenstine S, Wallace Z, Vlahov D. Urban built environment and depression: a multilevel analysis. J Epidemiol Community Health. 2005;59:822–7.

203. Gordon-Larsen P, Nelson MC, Page P, Popkin BM. Inequality in the built environment underlies key health disparities in physical activity and obesity. Pediatrics. 2006;117:417–24.

234. Grafova IB. Overweight children: Assessing the contribution of the built environment. Prev Med. 2008;47:304–8.

205. Heinrich KM, Lee RE, Regan GR, Reese-Smith JY, Howard HH, Haddock CK, et al. How does the built environment relate to body mass index and obesity prevalence among public housing residents? Am J Health Promot. 2008;22:187–94.

206. Lee SM, Tudor-Locke C, Burns EK. Application of a walking suitability assessment to the immediate built environment surrounding elementary schools. Health Promot Pract. 2008;9:246–52.

207. Lopez-Zetina J, Lee H, Friis R. The link between obesity and the built environment. Evidence from an ecological analysis of obesity and vehicle miles of travel in California. Health Place. 2006;12:656–64.

208. Wang G, Macera CA, Scudder-Soucie B, Schmid T, Pratt M, Buchner D, et al. Cost analysis of the built environment: the case of bike and pedestrian trials in Lincoln, Neb. Am J Public Health. 2004;94:549–53.

209. Van Holle V, Deforche B, Van Cauwenberg J, Goubert L, Maes L, Van de Weghe N, et al. Relationship between the physical environment and different domains of physical activity in European adults: a systematic review. BMC Public Health. 2012;12:807.

210. Stronegger WJ, Titze S, Oja P. Perceived characteristics of the neighborhood and its association with physical activity behavior and self-rated health. Health Place. 2010;16:736–43.

211. Titze S, Stronegger WJ, Janschitz S, Oja P. Environmental, social, and personal correlates of cycling for transportation in a student population. J Phys Act Health. 2007;4:66–79.

212. De Bourdeaudhuij I, Sallis JF, Saelens BE. Environmental correlates of physical activity in a sample of Belgian adults. Am J Health Promot. 2003;18:83–92.

213. Bourdeaudhuij ID, Teixeira PJ, Cardon G, Deforche B. Environmental and psychosocial correlates of physical activity in Portuguese and Belgian adults. Public Health Nutr Camb. 2005;8:886–95.

214. De Geus B, De Bourdeaudhuij I, Jannes C, Meeusen R. Psychosocial and environmental factors associated with cycling for transport among a working population. Health Educ Res. 2008;23:697–708.

215. Van Dyck D, Deforche B, Cardon G, De Bourdeaudhuij I. Neighbourhood walkability and its particular importance for adults with a preference for passive transport. Health Place. 2009;15:496–504.

216. Van Dyck D, Cardon G, Deforche B, De Bourdeaudhuij I. Urban–rural differences in physical activity in Belgian adults and the importance of psychosocial factors. J Urban Health. 2011;88:154–67.

217. Van Dyck D, Cardon G, Deforche B, Giles-corti B, Sallis JF, Owen N, et al. Environmental and psychosocial correlates of accelerometer-assessed and self-reported physical activity in Belgian adults. Int J Behav Med N Y. 2011;18:235–45.

218. Van Dyck D, Cardon G, Deforche B, De Bourdeaudhuij I. Do adults like living in high-walkable neighborhoods? Associations of walkability parameters with neighborhood satisfaction and possible mediators. Health Place. 2011;17:971–7.

219. Van Dyck D, Cardon G, Deforche B, Owen N, De Bourdeaudhuij I. Relationships between neighborhood walkability and adults’ physical activity: How important is residential self-selection? Health Place. 2011;17:1011–4.

220. Tuyckom CV. Macro-environmental factors associated with leisure-time physical activity: A cross-national analysis of EU countries. Scand J Public Health. 2011;39:419–26.

221. Vandenbulcke G, Thomas I, de Geus B, Degraeuwe B, Torfs R, Meeusen R, et al. Mapping bicycle use and the risk of accidents for commuters who cycle to work in Belgium. Transp Policy. 2009;16:77–87.

222. Vandenbulcke G, Dujardin C, Thomas I, Geus B de, Degraeuwe B, Meeusen R, et al. Cycle commuting in Belgium: Spatial determinants and ‘re-cycling’ strategies. Transp Res Part Policy Pract. 2011;45:118–37.

223. Milošević M, Golubić R, Mustajbegović J, Doko Jelinić J, Janev Holcer N, Kern J. Regional pattrn of physical inactivity in Croatia. Coll Antropol. 2009;33 Suppl 1:35–8.

224. Dygryn J, Mitas J, Stelzer J. The influence of built environment on walkability using geographic information system. J Hum Kinet. 2010;24:93–99.

225. Frömel K, Mitáš J, Kerr J. The associations between active lifestyle, the size of a community and SES of the adult population in the Czech Republic. Health Place. 2009;15:447–54.

226. Sigmundová D, El Ansari W, Sigmund E. Neighbourhood environment correlates of physical activity: a study of eight Czech regional towns. Int J Environ Res Public Health. 2011;8:341–57.

227. Toftager M, Ekholm O, Schipperijn J, Stigsdotter U, Bentsen P, Grønbœk M, et al. Distance to Green space and physical activity: a Danish national representative survey. J Phys Act Health. 2011;8:741–9.

228. Bertrais S, Preziosi P, Mennen L, Galan P, Hercberg S, Oppert J-M. Sociodemographic and geographic correlates of meeting current recommendations for physical activity in middle-aged French adults: the Supplémentation en Vitamines et Minéraux Antioxydants (SUVIMAX) Study. Am J Public Health. 2004;94:1560–6.

229. Pitsavos C, Panagiotakos DB, Lentzas Y, Stefanadis C. Epidemiology of leisure-time physical activity in socio-demographic, lifestyle and psychological characteristics of men and women in Greece: the ATTICA Study. BMC Public Health. 2005;5:37.

230. Bonnefoy XR, Braubach M, Moissonnier B, Monolbaev K, Röbbel N. Housing and health in Europe: preliminary results of a Pan-European Study. Am J Public Health. 2003;93:1559–63.

231. Engbers LH, Hendriksen IJ. Characteristics of a population of commuter cyclists in the Netherlands: perceived barriers and facilitators in the personal, social and physical environment. Int J Behav Nutr Phys Act. 2010;7:89.

232. Gast G-CM, Frenken FJM, van Leest LATM, Wendel-Vos GCW, Bemelmans WJE. Intra-national variation in trends in overweight and leisure time physical activities in the Netherlands since 1980: Stratification according to sex, age and urbanisation degree. Int J Obes. 2006;31:515–20.

233. Kamphuis C, Lenthe F, Giskes K, Huisman M, Brug J, Mackenbach J. Socio-economic status, social capital and sports participation. Med Sci Sports Exerc. 2008;40(1):71-81.

234. Keijer MJN, Rietveld P. How do people get to the railway station? The Dutch experience. Transp Plan Technol. 2000;23:215–35.

235. Maas J, Verheij RA, Spreeuwenberg P, Groenewegen PP. Physical activity as a possible mechanism behind the relationship between green space and health: A multilevel analysis. BMC Public Health. 2008;8:206.

236. Maat K, Timmermans HJP. Influence of the residential and work environment on car use in dual-earner households. Transp Res Part Policy Pract. 2009;43:654–64.

237. van Lenthe FJ, Brug J, Mackenbach JP. Neighbourhood inequalities in physical inactivity: the role of neighbourhood attractiveness, proximity to local facilities and safety in the Netherlands. Soc Sci Med. 2005;60:763–75.

238. Wendel-Vos GCW, Schuit AJ, De Niet R, Boshuizen HC, Saris WHM, Kromhout D. Factors of the physical environment associated with walking and bicycling. Med Sci Sports Exerc. 2004;36:725–30.

239. Wendel-Vos GCW, Hooijdonk C van, Uitenbroek D, Agyemang C, Lindeman EM, Droomers M. Environmental attributes related to walking and bicycling at the individual and contextual level. J Epidemiol Community Health. 2008;62:689–94.

240. Kwaśniewska M, Kaczmarczyk-Chałas K, Pikala M, Broda, Kozakiewicz K, Pająk A, et al. Socio-demographic and lifestyle correlates of commuting activity in Poland. Prev Med. 2010;50:257–61.

241. Santana P, Santos R, Nogueira H. The link between local environment and obesity: A multilevel analysis in the Lisbon metropolitan area, Portugal. Soc Sci Med. 2009;68:601–9.

242. Rute Santos MS, Susana Vale MS, Miranda L, Mota J. Socio-demographic and perceived environmental correlates of walking in Portuguese adults—A multilevel analysis. Health & Place. 2009;15:1094–9.

243. Santos R, Silva P, Santos P, Ribeiro JC, Mota J. Physical activity and perceived environmental attributes in a sample of Portuguese adults: Results from the Azorean Physical Activity and Health Study. Prev Med. 2008;47:83–8.

244. Bolívar J, Daponte A, Rodríguez M, Sánchez JJ. The influence of individual, social and physical environment factors on physical activity in the adult population in Andalusia, Spain. Int J Environ Res Public Health. 2010;7:60–77.

245. Molina-García J, Castillo I, Sallis JF. Psychosocial and environmental correlates of active commuting for university students. Prev Med. 2010;51:136–8.

246. Pascual C, Regidor E, Martínez D, Elisa Calle M, Domínguez V. Socioeconomic environment, availability of sports facilities, and jogging, swimming and gym use. Health Place. 2009;15:553–61.

247. Bergman P, Grjibovski AM, Hagströmer M, Bauman A, Sjöström M. Adherence to physical activity recommendations and the influence of socio-demographic correlates – a population-based cross-sectional study. BMC Public Health. 2008;8:367.

248. Bergman P, Grjibovski AM, Hagströmer M, Sallis JF, Sjöström M. The association between health enhancing physical activity and neighbourhood environment among Swedish adults – a population-based cross-sectional study. Int J Behav Nutr Phys Act. 2009;6:8.

249. Björk J, Albin M, Grahn P, Jacobsson H, Ardö J, Wadbro J, et al. Recreational values of the natural environment in relation to neighbourhood satisfaction, physical activity, obesity and wellbeing. J Epidemiol Community Health. 2008;62:e2–e2.

250. Gidlöf-Gunnarsson A, Öhrström E. Noise and well-being in urban residential environments: The potential role of perceived availability to nearby green areas. Landsc Urban Plan. 2007;83:115–26.

251. Sundquist K, Eriksson U, Kawakami N, Skog L, Ohlsson H, Arvidsson D. Neighborhood walkability, physical activity, and walking behavior: The Swedish Neighborhood and Physical Activity (SNAP) study. Soc Sci Med. 2011;72:1266–73.

252. Cochrane T, Davey RC, Gidlow C, Smith GR, Fairburn J, Armitage CJ, et al. Small area and individual level predictors of physical activity in urban communities: a multi-level study in Stoke on Trent, England. Int J Environ Res Public Health. 2009;6:654–77.

253. Foster C, Hillsdon M, Thorogood M. Environmental perceptions and walking in English adults. J Epidemiol Community Health. 2004;58:924–8.

254. Foster C, Hillsdon M, Jones A, Grundy C, Wilkinson P, Wareham N, et al. Objective measures of the environment and physical activity : results of the environment and physical activity study in English adults. J Phys Act Health. 2009;Vol.6 Suppl.1:S70–80.

255. Foster CE, Panter JR, Wareham NJ. Assessing the impact of road traffic on cycling for leisure and cycling to work. Int J Behav Nutr Phys Act. 2011;8:61.

256. Harrison RA, Gemmell I, Heller RF. Evidence based public health policy and practice: The population effect of crime and neighbourhood on physical activity: an analysis of 15461 adults. J Epidemiol Community Health 1979-. 2007;61:34–9.

257. Hillsdon M, Panter J, Foster C, Jones A. The relationship between access and quality of urban green space with population physical activity. Public Health. 2006;120:1127–32.

258. Jones A, Hillsdon M, Coombes E. Greenspace access, use, and physical activity: Understanding the effects of area deprivation. Prev Med. 2009;49:500–5.

259. Mason P, Kearns A, Bond L. Neighbourhood walking and regeneration in deprived communities. Health Place. 2011;17:727–37.

260. Ogilvie D, Mitchell R, Mutrie N, Petticrew M, Platt S. Personal and environmental correlates of active travel and physical activity in a deprived urban population. Int J Behav Nutr Phys Act. 2008;5:43.

261. Panter J, Jones A, Hillsdon M. Equity of access to physical activity facilities in an English city. Prev Med. 2008;46:303–7.

262. Panter JR, Jones AP. Associations between physical activity, perceptions of the neighbourhood environment and access to facilities in an English city. Soc Sci Med. 2008;67:1917–23.

263. Panter J, Jones A, van Sluijs E, Griffin S, Wareham N. Environmental and psychological correlates of older adult’s active commuting. Med Sci Sports Exerc. 2011; doi:10.1249/MSS.0b013e3182078532.

264. Parkes A, Kearns A. The multi-dimensional neighbourhood and health: a cross-sectional analysis of the Scottish Household Survey, 2001. Health Place. 2006;12:1–18.

265. Parkin J, Wardman M, Page M. Estimation of the determinants of bicycle mode share for the journey to work using census data. Transp N Y. 2008;35:93–109.

266. Stafford M, Cummins S, Ellaway A, Sacker A, Wiggins RD, Macintyre S. Pathways to obesity: Identifying local, modifiable determinants of physical activity and diet. Soc Sci Med. 2007;65:1882–97.

267. Bamana A, Tessier S, Vuillemin A. Association of perceived environment with meeting public health recommendations for physical activity in seven European countries. J Public Health. 2008;30:274–81.

268. Ellaway A, Macintyre S, Bonnefoy X. Graffiti, greenery, and obesity in adults: secondary analysis of European cross sectional survey. BMJ. 2005;331:611–2.

269. Guthold R, Ono T, Strong KL, Chatterji S, Morabia A. Worldwide variability in physical inactivity: A 51-Country survey. Am J Prev Med. 2008;34:486–94.

270. Miles R. Neighborhood disorder, perceived safety, and readiness to encourage use of local playgrounds. Am J Prev Med. 2008;34:275–81.

271. Rütten A, Abel T, Kannas L, Lengerke T von, Lüschen G, Diaz JAR, et al. Self reported physical activity, public health, and perceived environment: results from a comparative European study. J Epidemiol Community Health. 2001;55:139–46.

272. Rütten A, Abu-Omar K. Perceptions of environmental opportunities for physical activity in the European Union. Soz- Präventivmedizin Soc Prev Med Médecine Soc Préventive. 2004;49:310–7.

273. Shenassa ED, Liebhaber A, Ezeamama A. Perceived safety of area of residence and exercise: a Pan-European study. Am J Epidemiol. 2006;163:1012–7.

274. Ståhl T, Rütten A, Nutbeam D, Bauman A, Kannas L, Abel T, et al. The importance of the social environment for physically active lifestyle — results from an international study. Soc Sci Med. 2001;52:1–10.

275. Zapata-Diomedi B, Veerman JL. The association between built environment features and physical activity in the Australian context: a synthesis of the literature. BMC Public Health. 2016;16:484.

276. Astell-Burt T, Feng X, Kolt GS. Green space is associated with walking and Moderate-to-Vigorous Physical Activity (MVPA) in middle-to-older-aged adults: findings from 203883 Australians in the 45 and Up Study. Br J Sports Med. 2014;48:404–6.

277. Astell-Burt T, Feng X, Kolt GS. Identification of the impact of crime on physical activity depends upon neighbourhood scale: Multilevel evidence from 203883 Australians. Health Place. 2015;31 Suppl C:120–3.

278. Christian HE, Bull FC, Middleton NJ, Knuiman MW, Divitini ML, Hooper P, et al. How important is the land use mix measure in understanding walking behaviour? Results from the RESIDE study. Int J Behav Nutr Phys Act. 2011;8:55.

279. Cleland VJ, Ball K, Crawford D. Is a perceived supportive physical environment important for self-reported leisure time physical activity among socioeconomically disadvantaged women with poor psychosocial characteristics? An observational study. BMC Public Health. 2013;13:280.

280. Duncan MJ, Winkler E, Sugiyama T, Cerin E, duToit L, Leslie E, et al. Relationships of land use mix with walking for transport: Do land uses and geographical scale matter? J Urban Health Bull N Y Acad Med. 2010;87:782–95.

281. Foster S, Knuiman M, Villanueva K, Wood L, Christian H, Giles-Corti B. Does walkable neighbourhood design influence the association between objective crime and walking? International Journal of Behavioral Nutrition and Physical Activity. 2014;11:100.

282. Giles-Corti B, Bull F, Knuiman M, McCormack G, Van Niel K, Timperio A, et al. The influence of urban design on neighbourhood walking following residential relocation: Longitudinal results from the RESIDE study. Soc Sci Med. 2013;77 Suppl C:20–30.

283. Heesch KC, Giles-Corti B, Turrell G. Cycling for transport and recreation: Associations with socio-economic position, environmental perceptions, and psychological disposition. Prev Med. 2014;63 Suppl C:29–35.

284. Knuiman MW, Christian HE, Divitini ML, Foster SA, Bull FC, Badland HM, et al. A longitudinal analysis of the influence of the neighborhood built environment on walking for transportation: The RESIDE Study. Am J Epidemiol. 2014;180:453–61.

285. Koohsari MJ, Kaczynski AT, Giles-Corti B, Karakiewicz JA. Effects of access to public open spaces on walking: Is proximity enough? Landsc Urban Plan. 2013;117 Suppl C:92–9.

286. Koohsari MJ, Karakiewicz JA, Kaczynski AT. Public open space and walking: the role of proximity, perceptual qualities of the surrounding built environment, and street configuration. Environ Behav. 2013;45:706–36.

287. Koohsari MJ, Sugiyama T, Lamb KE, Villanueva K, Owen N. Street connectivity and walking for transport: Role of neighborhood destinations. Prev Med. 2014;66 Suppl C:118–22.

288. Learnihan V, VAN NIEL KP, GILES‐CORTI B, KNUIMAN M. Effect of scale on the links between walking and urban design. Geogr Res. 2011;49:183–91.

289. McCormack GR, Shiell A, Giles-Corti B, Begg S, Veerman JL, Geelhoed E, et al. The association between sidewalk length and walking for different purposes in established neighborhoods. Int J Behav Nutr Phys Act. 2012;9:92.

290. McKibbin M: The influence of the built environment on mode choice – evidence from the journey to work in Sydney. World Transit Res. 2011. http://www.worldtransitresearch.info/research/4346. Accessed Feb 2 2018.

291. Shimura H, Sugiyama T, Winkler E, Owen N. High neighborhood walkability mitigates declines in middle-to-older aged adults’ walking for transport. J Phys Act Health. 2012;9:1004–8.

292. Sugiyama T, Cerin E, Owen N, Oyeyemi AL, Conway TL, Van Dyck D, et al. Perceived neighbourhood environmental attributes associated with adults׳ recreational walking: IPEN Adult study in 12 countries. Health Place. 2014;28 Suppl C:22–30.

293. Sugiyama T, Giles-Corti B, Summers J, du Toit L, Leslie E, Owen N. Initiating and maintaining recreational walking: A longitudinal study on the influence of neighborhood green space. Prev Med. 2013;57:178–82.

294. Sugiyama T, Leslie E, Giles-Corti B, Owen N. Physical activity for recreation or exercise on neighbourhood streets: Associations with perceived environmental attributes. Health Place. 2009;15:1058–63.

295. Titze S, Giles-Corti B, Knuiman MW, Pikora TJ, Timperio A, Bull FC, et al. Associations between intrapersonal and neighborhood environmental characteristics and cycling for transport and recreation in adults: baseline results from the RESIDE Study. J Phys Act Health. 2010;7:423–31.

296. Wilson KM, Brady TJ, Lesesne C, Translation NWG. An organizing framework for translation in public health: the knowledge to action framework. Prev Chronic Dis. 2011;8:A46.
